# Multi-Omics Integrated Analysis Reveals Correlative Signatures of Short-Chain PFAS Mixtures on Mouse Midbrain Dopaminergic Neurons Involving the TM/5-HT Pathway

**DOI:** 10.3390/ijms27104543

**Published:** 2026-05-19

**Authors:** Tianao Sun, Minli Yang, Yongjie Ma, Zhanyue Zheng, Jinhao Wan, Jingxia Wei, Minglian Pan, Yingjie Zhou, Xinyu Yuan, You Li, Yan Sun

**Affiliations:** 1 School of Public Health, Guilin Medical University, Guilin 541199, China; 17791635922@163.com (T.S.); 18897732730@163.com (M.Y.); mayongjie0606@163.com (Y.M.); zhengzhanyue999@163.com (Z.Z.); wjhwio@163.com (J.W.); m18777893571@163.com (J.W.); pml18278456264@163.com (M.P.); 15221582965@163.com (Y.Z.); 18285094298@163.com (X.Y.); liyou121300@163.com (Y.L.); 2 Guangxi Key Laboratory of Environmental Exposomics and Entire Lifecycle Health, Guilin Medical University, Guilin 541199, China

**Keywords:** PFAS, DA neuronal damage, transcriptome sequencing, metabolomics, 16S rRNA analysis

## Abstract

This study aimed to identify candidate molecular pathways mediating dopaminergic dysfunction induced by PFAS mixture exposure, with a focus on the TM/5-HT signaling axis and calcium-linked lipid metabolites, and to explore potential gut-brain axis involvement. Adult mice were exposed to a PFAS mixture. Behavioral tests assessed spatial memory, spontaneous activity, and motor coordination. Histopathological and ultrastructural analyses examined neuronal atrophy, mitochondrial damage, α-synuclein (α-syn), and tyrosine hydroxylase (TH). Transcriptomics, metabolomics, and gut microbiota profiling (16S rRNA sequencing) were performed, followed by integrated multi-omics and correlation analyses. PFAS exposure was associated with PD-relevant motor and cognitive impairments, including impaired spatial memory, reduced spontaneous activity, and motor coordination deficits. Neuronal atrophy, mitochondrial structural damage, upregulation of α-syn, and downregulation of TH were observed. Transcriptomics identified 315 differentially expressed genes (DEGs) enriched in ciliary movement, neuroactive ligand-receptor interactions, and serotonergic synapses. Metabolomics identified 130 differentially abundant metabolites involved in arachidonic acid metabolism and serotonergic synapses. Integrated analysis highlighted correlative changes in the TM/5-HT signaling pathway. Phosphatidylinositol PI(16:0/20:2(11Z,14Z)) showed a strong positive correlation with *Dbh* gene expression, suggesting a candidate association between *Dbh* expression and phosphatidylinositol alterations. Gut microbiota analysis revealed compositional alterations (e.g., *Muribaculaceae*, *Ileibacterium*) and predicted functional shifts (e.g., tryptophan metabolism–related modules) were observed; these findings are exploratory. This study identifies multi-omics signatures associated with PFAS mixture-induced dopaminergic dysfunction in mice. The TM/5-HT pathway emerges as a candidate molecular axis requiring further investigation. Gut microbiota alterations suggest a potential peripheral component, but causality and gut-brain axis involvement remain hypothetical and need direct experimental validation.

## 1. Introduction

These PFAS have been applied in a wide range of consumer products as important and common additives since the 1950s, including packaging bags, waterproof fabrics, waxes, fire-fighting foams, shampoos and cosmetics, and pesticides [1]. However, many PFAS have been shown to be metabolically toxic, immunotoxic, neurotoxic, and embryotoxic in recent years [2,3,4,5,6]. They can contaminate the water and food through direct discharge, migration, enrichment, and amplification in the marine ecosystems [7]. PFASs exhibit high bioaccumulation potential in organisms and intrinsic toxicity that poses significant health risks [8]. Additionally, they undergo long-range environmental transport via air and water currents. Their degradation is extremely slow in natural environments, primarily attributed to the stable perfluorinated carbon chains [9,10].

PFAS are primarily classified into two categories: short-chain and long-chain compounds. Globally, the most frequently monitored PFAS are perfluoroalkyl acids (PFAAs), further subcategorized as perfluorocarboxylic acids (PFCAs) and perfluorosulfonic acids (PFSAs). The prototypical representatives of these subclasses are perfluorooctanoic acid (PFOA) and perfluorooctanesulfonic acid (PFOS), respectively [11]. In recent years, specific long-chain perfluoroalkyl substances, notably PFOA and PFOS, have been formally listed under the Stockholm Convention on Persistent Organic Pollutants. Following the widespread phase-out of long-chain PFAS, short-chain PFAS have gained increasing attention as proposed alternatives to address the persistent environmental contamination and associated human exposure risks stemming from legacy long-chain compounds [12]. Wang et al. [13] analyzed PFAS concentrations in Chinese surface waters between 2010 and 2020, documenting a compositional shift from dominant PFOA and PFOS toward prevalent short-chain congeners, including perfluorobutanoate (PFBA), perfluorobutanesulfonate (PFBS), perfluorohexanoate (PFHxA), perfluorohexanesulfonate (PFHxS), and perfluoropentanoate (PFPeA). Concurrently, industrial adoption of presumptive lower-toxicity alternatives has increased, resulting in elevated environmental emissions [14,15,16,17]. Scientific monitoring reveals widespread occurrence of short-chain per- and PFAS in global surface waters and aquatic ecosystems [18]. Owing to their physicochemical properties, these compounds present persistent analytical challenges for accurate identification using conventional methodologies, thereby heightening concerns regarding their ecological and human health implications [19,20].

Exposure to PFAS mixtures is associated with neurotoxic effects, such as behavioral abnormalities and cognitive deficits, potentially involving neurotransmitter-related processes and cellular stress pathways. Dietary exposure to environmentally relevant PFAS mixtures significantly decreased midbrain dopamine levels and downregulated tyrosine hydroxylase (TH) expression, the rate-limiting enzyme for dopamine biosynthesis, in male mice. In brain tissues, perfluorocarboxylates (PFCAs, PFBA, and PFHxA) and perfluorosulfonates (PFSAs, PFBS, and PFOS) constitute the predominant detectable forms with the highest concentrations [21]. Experimental studies suggest PFBS and PFBA exhibit potential organ-specific toxicity, while supraphysiological concentrations of PFHxA disrupt neurotransmission and suppress neuroexcitation in the central nervous system [22]. Humans are chronically exposed to complex mixtures of environmentally persistent contaminants through concurrent multipathway exposure routes. Due to potential mixture interactions (additive, synergistic, or antagonistic effects) among co-occurring PFAS, combined exposures at individually subthreshold concentrations may induce eco-toxicological outcomes of concern despite constituent levels below single-substance effect thresholds [23].

This study aims to characterize the neurotoxic consequences of combined exposure to PFBS, PFBA, and PFHxA on mouse midbrain dopaminergic neurons using a multi-omics approach (transcriptomics, metabolomics, and gut microbiota 16S rRNA analysis). Rather than establishing definitive mechanisms or therapeutic targets, we seek to identify correlative molecular pathways and candidate gene-metabolite networks that may generate testable hypotheses for future mechanistic studies. The inclusion of gut microbiota analysis is exploratory, aiming to assess potential peripheral contributions via the gut-brain axis.

## 2. Results

### 2.1. Behavioral Results

#### PFAS Mixture Exposure Induces Multiple Behavioral Deficits Resembling Parkinsonian Phenotypes

To assess whether sub-chronic exposure to the PFAS mixture (PFBS + PFBA + PFHxA) produces behavioral alterations relevant to dopaminergic dysfunction, we performed a battery of motor and cognitive tests.

**Figure 1:**
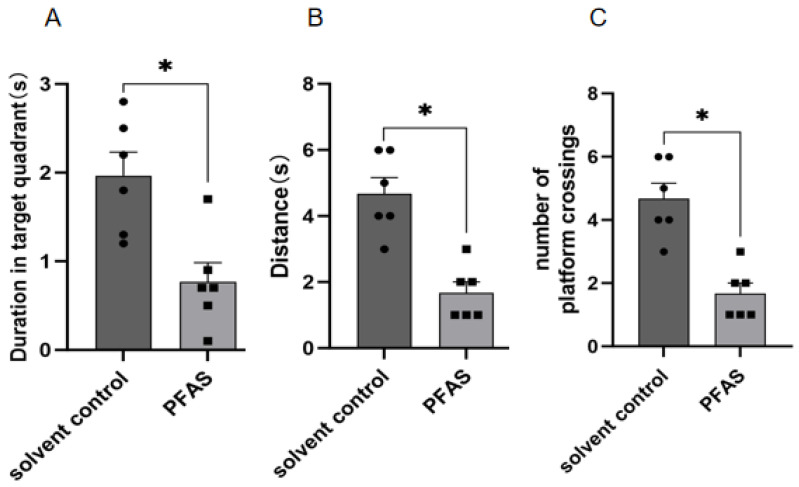
PFAS mixture exposure impairs spatial memory in the Morris water maze probe trial. (**A**) Time spent in the target quadrant (seconds); (**B**) Total distance traveled in the target quadrant (cm); (**C**) Number of platform crossings. Six mice were used in each group (solvent control: *n* = 6; PFAS-exposed: *n* = 6). Statistical analysis was performed using Student’s *t*-test “*” indicating *p* < 0.05.

Spatial memory deficits. Using the Morris water maze probe trial, PFAS-exposed mice showed significant impairments in spatial learning and memory compared to vehicle controls. As shown in Figure 1, exposed mice spent less time in the target quadrant (*p* < 0.05), swam shorter distances (*p* < 0.05), and exhibited fewer platform crossings (*p* < 0.05). These findings indicate that PFAS mixture exposure compromises hippocampal-dependent spatial memory.

**Figure 2:**
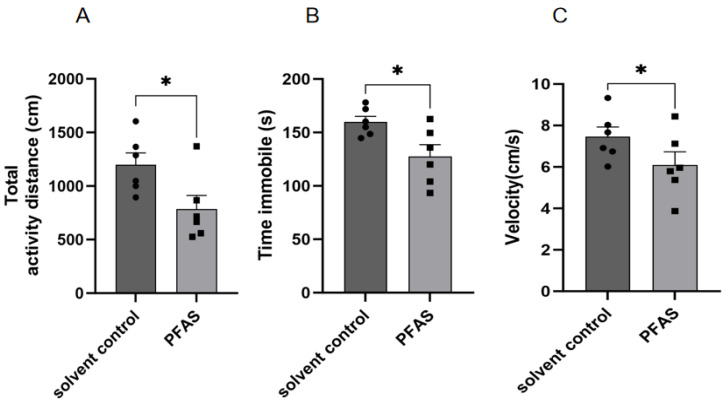
Results of open field experiment (**A**) Total activity distance (cm); (**B**) Total time of exercise; (**C**) Average speed; “*”, indicating *p* < 0.05 compared with the Solvent control group. Six mice were used in each group (solvent control: *n* = 6; PFAS-exposed: *n* = 6). Statistical analysis was performed using Student’s *t*-test.

Reduced spontaneous locomotion and exploratory behavior. In the open field test, PFAS-exposed mice exhibited significantly decreased total distance traveled, reduced average speed, and lower ambulatory time percentage relative to controls (Figure 2, all *p* < 0.05). No anxiety-like behaviors (e.g., reduced center time) were observed, suggesting that the reduced locomotion reflects motor slowing rather than emotional changes.

**Figure 3:**
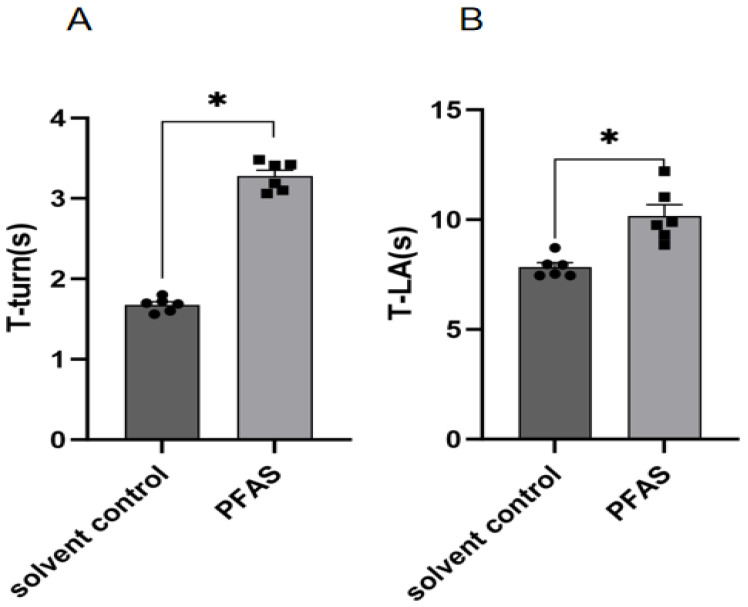
Results of pole test. (**A**) Turning time; (**B**) Total duration; “*”, indicating *p* < 0.05 compared with the Solvent control group. Six mice were used in each group (solvent control: *n* = 6; PFAS-exposed: *n* = 6). Statistical analysis was performed using Student’s *t*-test.

Impaired motor coordination. The pole test, which measures bradykinesia and motor coordination, revealed that PFAS-exposed mice required significantly more time to turn downward (T-turn) and to descend the entire pole (T-LA) compared to controls (Figure 3, *p* < 0.05 for both).

**Figure 4:**
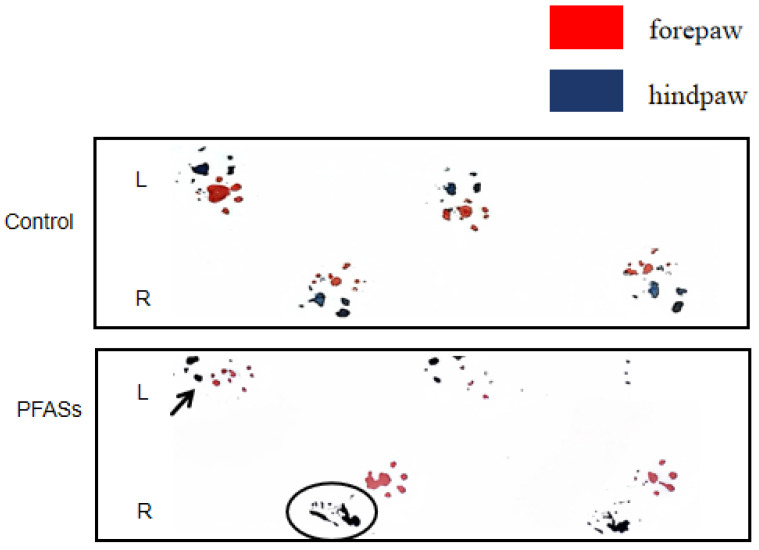
Mouse footprint map. Footprint impressions of the control (**above**) and PFAS (**below**). Smearing artifacts due to dragging are marked with circles, and indistinct footprints are indicated with arrows. ‘L’ stands for left foot, and ‘R’ stands for right foot.

**Figure 5:**
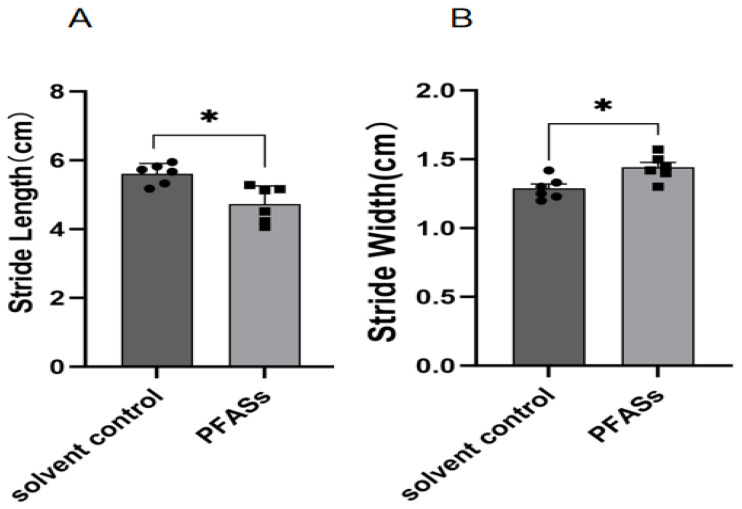
Gait analysis experiment. (**A**) Stride Length; (**B**) Stride Width; “*”, indicating *p* < 0.05 compared with the Solvent control group. Six mice were used in each group (solvent control: *n* = 6; PFAS-exposed: *n* = 6). Statistical analysis was performed using Student’s *t*-test.

Gait abnormalities. Quantitative gait analysis (Figure 4 and Figure 5) demonstrated that PFAS-exposed mice exhibited wider step width and shorter stride length relative to controls (*p* < 0.05). Representative footprint patterns showed incomplete plantar registration in the left hindpaw (arrowed) and a persistent ink trail in the right hindlimb (circled), indicative of impaired interlimb coordination and neuromuscular dysfunction.

Collectively, these behavioral results demonstrate that exposure to the PFAS mixture recapitulates multiple parkinsonism-related features, including spatial memory deficits, hypokinesia, motor incoordination, and gait disturbances. Having established these behavioral phenotypes, we next examined the underlying pathological and molecular alterations in the midbrain.

### 2.2. Pathological Changes

#### 2.2.1. Co-Exposure to PFAS and the Morphological Characteristics of the Midbrain Dopaminergic Neurons

Compared to solvent controls, PFAS-exposed mouse midbrain tissues exhibited significant neuronal atrophy (indicated by black arrows, Supporting Information Figure S1) and a significantly elevated proportion of neurons displaying atrophic morphology.

#### 2.2.2. Co-Exposure to PFAS Alterations in Midbrain Nigrostriatal TH+ Neurons

The Support Information Figure S2 shows compared to solvent control mice, midbrain nigrostriatal dopaminergic neurons (TH+) in PFAS-exposed mice exhibited reduced TH immunoreactivity intensity. However, stereological quantification revealed no significant reduction in the number of TH+ neurons within the substantia nigra pars compacta (SNpc) (*p* > 0.05).

#### 2.2.3. Co-Exposure to PFAS Damage to the Ultrastructure of Midbrain Dopaminergic Neurons

Compared to solvent controls, PFAS-exposed mouse neuronal cells exhibited mild intracellular edema while maintaining plasma membrane integrity. Transmission electron microscopy revealed a reduction in cytoplasmic electron density accompanied by mild organellar swelling in PFAS-exposed neurons compared to solvent controls. TEM analysis revealed rounded nuclear contours with an ill-defined nuclear envelope in PFAS-exposed neurons. Mitochondrial ultrastructure in PFAS-exposed neurons exhibited significant pathology: moderate dilatation, rarefied matrix with heterogeneous density distribution, disrupted and diminished cristae, intramitochondrial myelin figures, and focal membrane fragmentation (Support Information Figure S3, red arrowheads). These findings indicate structural damage but do not directly demonstrate functional impairment or mitophagy.

### 2.3. Co-Exposure to PFAS Reduces Expression Levels of DA Neuron-Specific Markers in the Midbrain

**Figure 6:**
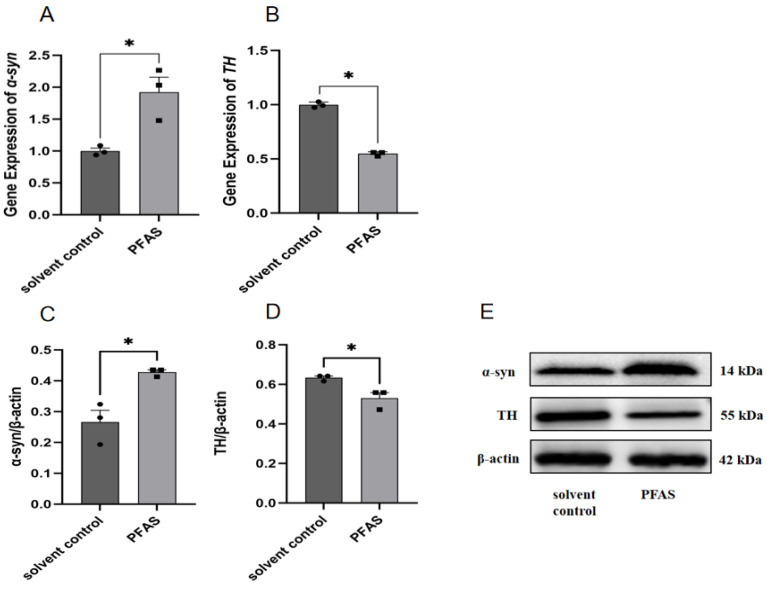
Expression levels of α-synuclein (α-syn) and tyrosine hydroxylase (TH) in mouse midbrain. Note: (**A**) The relative expression of α-syn mRNA; (**B**) The relative expression of TH mRNA; (**C**) α-syn protein expression level; (**D**) TH protein expression level; (**E**) WB strip chart of α-syn and TH; “*”, indicating *p* < 0.05 compared with the Solvent control group. Three mice were used in each group (solvent control: *n* = 3; PFAS-exposed: *n* = 3). Statistical analysis was performed using Student’s *t*-test.

Midbrain α-syn expression was significantly upregulated at both mRNA and protein levels in PFAS-exposed mice compared to solvent controls (*p* < 0.05; Figure 6A,B). This indicates increased total α-syn expression; however, we did not directly assess α-syn aggregation or oligomerization. Conversely, TH expression showed significant downregulation at both transcriptional (mRNA) and protein levels (*p* < 0.05; Figure 6C,D).

### 2.4. Co-Exposure to PFAS on Differential Gene Expression Profiles in Midbrain DA Neurons

RNA sequencing was performed to profile transcriptional alterations in the mouse midbrain following combined exposure to PFAS.

#### 2.4.1. Differential Expression Gene Screening

To further explore the cause of midbrain DA neuronal damage in mice exposed to a mixture of PFAS. Transcriptomic analysis identified 315 DEGs in PFAS-exposed midbrains compared to solvent controls (FDR < 0.05, |log_2_FC| ≥ 1.5), with 57 significantly upregulated and 258 downregulated genes (Support Information Figure S4).

#### 2.4.2. Functional Enrichment Analysis of Differentially Expressed Genes

**Figure 7:**
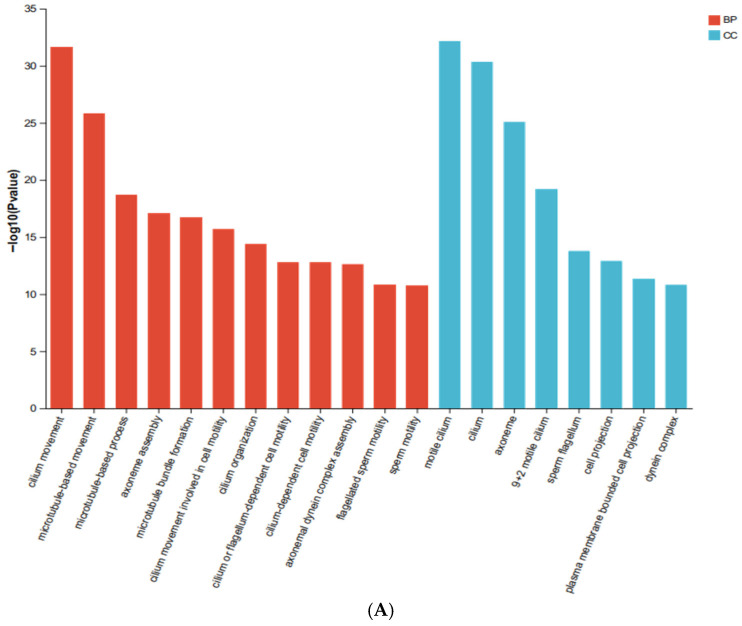

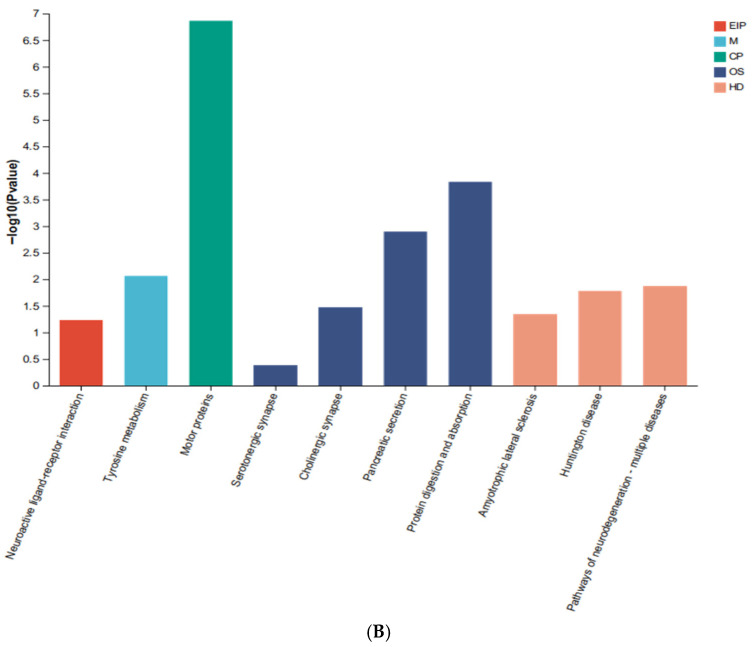
Functional enrichment analysis of down-regulated differentially expressed genes. (**A**) Gene Ontology (GO) enrichment analysis. Two colors represent the two main categories: biological processes (BP) and cellular components (CC). Only terms with FDR < 0.05 are shown. (**B**) Kyoto Encyclopedia of Genes and Genomes (KEGG) pathway enrichment analysis. Different colors represent branches of KEGG pathways: environmental information processing (EIP), metabolism (M), cellular processes (CP), organic systems (OS), and human diseases (HD). Only pathways with FDR < 0.05 are shown. Statistical analysis: Differential expression was performed using DESeq2. DEGs were defined as FDR < 0.05 and |log_2_FC| ≥ 1.5. Enrichment analysis was performed using hypergeometric test with Benjamini-Hochberg correction. Sample size: *n* = 3 mice per group (solvent control: *n* = 3; PFAS-exposed: *n* = 3).

GO enrichment analysis (Figure 7A) revealed that PFAS exposure primarily affected genes involved in cilium-related processes. Specifically, downregulated DEGs were enriched in biological processes such as “cilium movement” and “microtubule-based movement,” and cellular components such as “motile cilium” and “dynein complex” (FDR < 0.05). Biological interpretation: Primary cilia on dopaminergic neurons act as signaling hubs for dopamine receptors and are critical for neuronal survival and function. Disruption of ciliary genes by PFAS may therefore impair dopamine signaling even before overt neuronal loss occurs.

KEGG pathway analysis (Figure 7B) identified three neurotransmitter-related pathways among the downregulated DEGs: neuroactive ligand-receptor interaction, tyrosine metabolism, and serotonergic synapse (FDR < 0.05). Biological interpretation: Tyrosine metabolism is the primary source of dopamine precursors; downregulation of genes in this pathway (e.g., *Dbh*, *Tyrp1*) suggests reduced capacity for dopamine synthesis. Concurrent disruption of serotonergic synapse genes (e.g., *Htr5b*, *Gng8*) indicates broader monoaminergic dysfunction, which may explain the motor and cognitive deficits observed in behavioral tests.

#### 2.4.3. Signaling Pathway Analysis and Key Gene Screening

This study specifically investigated the tryptophan hydroxylase (TPH)/5-HT pathway due to its established role in dopaminergic neuron pathophysiology, aligning with our research focus on PFAS mixture-induced damage to midbrain dopaminergic neurons. Dysregulation of the TPH/5-HT pathway compromises dopamine biosynthesis, directly contributing to dopaminergic dysfunction. Furthermore, disrupted serotonergic neurotransmission indirectly impairs neuronal excitability and accelerates neurodegenerative pathology in dopaminergic systems. Transcriptomic analysis revealed significant enrichment of DEGs in key pathways: TPH pathway: *Dbh*, *Tyrp1*, *Got1l1*; 5-HT signaling pathway: *Gng8*, *Htr5b*; Corresponding visualization in Support Information Figure S5. We verified the expression of *Dbh*, *Tyrp1*, *Got1l1* and *Gng8*. Quantitative PCR analysis revealed significantly reduced mRNA expression of *Dbh*, *Tyrp1*, *Got1l1*, and *Gng8* in PFAS-exposed mice versus solvent controls. *Dbh* exhibited the most pronounced downregulation (Support Information Figure S6) concordant with transcriptomic profiling data.

#### 2.4.4. Protein Interaction Network Analysis and Hub Gene Screening

**Figure 8:**
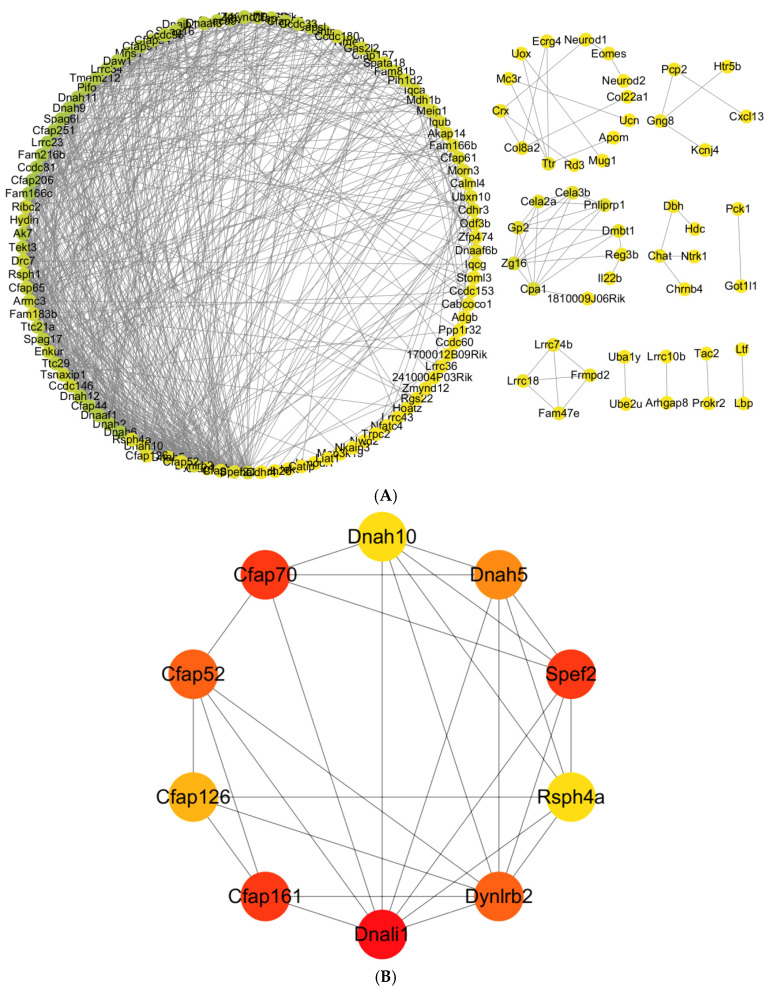
Protein-protein interaction (PPI) network analysis of differentially expressed genes. (**A**) PPI network of 315 DEGs. Nodes represent proteins encoded by DEGs; edges represent known or predicted interactions (confidence score ≥ 0.4). The network was constructed using STRING database (v11.5) and visualized in Cytoscape (v3.9.1). (**B**) Top 10 hub genes identified by degree centrality using the cytoHubba plugin. Darker colors indicate higher node degree (more connections). Statistical analysis: Hub genes were ranked by degree centrality. The network includes 153 nodes and 699 edges. Only interactions with STRING combined score > 0.4 are shown. Sample size: *n* = 3 mice per group (solvent control: *n* = 3; PFAS-exposed: *n* = 3).

Protein-protein interaction (PPI) network reconstruction was performed using the STRING database (v11.5) to visualize interactions among DEG-encoded proteins and to identify hub genes within the network. Utilizing STRING-db’s functional module, a PPI network was generated for the 315 DEGs, yielding 153 nodes and 699 edges with significant interconnectivity (Figure 8A). The 153 network nodes represent protein products encoded by the DEGs, indicating functional interactions among 48.6% (153/315) of the identified DEGs. The 699 high-confidence edges reflect intricate protein interactivity, with nodes forming cooperative complexes or regulatory relationships that collectively orchestrate key cellular functions. Using the cytoHubba plugin in Cytoscape v3.9.1, the top 10 hub genes by degree centrality were Dnali1 (degree = 44), Spef2 (degree = 33), Cfap70 (degree = 33), Cfap161 (degree = 32), Dynlrb2 (degree = 31), Dnah5 (degree = 29), Rsph4a (degree = 28), Cfap126 (degree = 27), and Dnah10 (degree = 26) (Figure 8B). These hub genes are primarily involved in cilium structure and function, consistent with our GO enrichment analysis.

#### 2.4.5. Functional Annotation Analysis of Hub Genes

**Figure 9:**
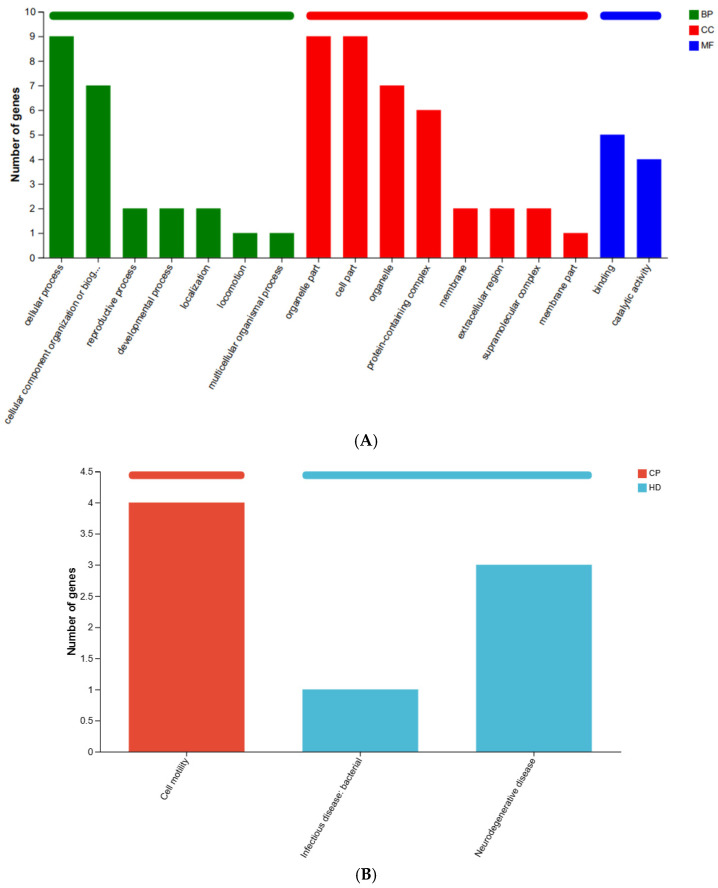
Functional annotation analysis of DEGs. (**A**) GO functional annotation analysis. Three colors represent the three main categories: biological processes (BP), cellular components (CC), and molecular functions (MF). Terms with FDR < 0.05 are shown. (**B**) KEGG pathway annotation analysis. Different colors represent KEGG pathway branches: cellular processes (CP) and human diseases (HD). Only pathways with FDR < 0.05 are shown. Statistical analysis: Enrichment analysis was performed using clusterProfiler with Benjamini-Hochberg correction for multiple testing (FDR < 0.05). Sample size: *n* = 3 mice per group (solvent control: *n* = 3; PFAS-exposed: *n* = 3).

GO functional annotation analysis (Figure 9A) found functional annotations of DEGs focused on multicellular biological processes, motility, localization, developmental processes, reproductive processes, organization of cellular components or biogenesis, cellular processes, membrane parts, supramolecular complexes, extracellular regions, membranes, protein-containing complexes, organelles, cellular parts, organelle parts, catalytic activity, and binding. KEGG Pathway Annotation Analysis (Figure 9B) revealed that the pathway is enriched in cell motility, infectious diseases, and neurodegenerative diseases.

### 2.5. Co-Exposure to PFAS Analysis of Characteristic Changes in the Metabolome

#### 2.5.1. Differential Metabolite Statistics

Differentially abundant metabolites were identified by integrating variable importance in projection values derived from orthogonal partial least squares-discriminant analysis with statistical significance thresholds (Support Information Figure S7). Metabolomic profiling identified 130 significantly dysregulated metabolites, comprising 39 up-regulated and 91 down-regulated species (Support Information Figure S8).

#### 2.5.2. KEGG Pathway Enrichment Analysis

**Figure 10:**
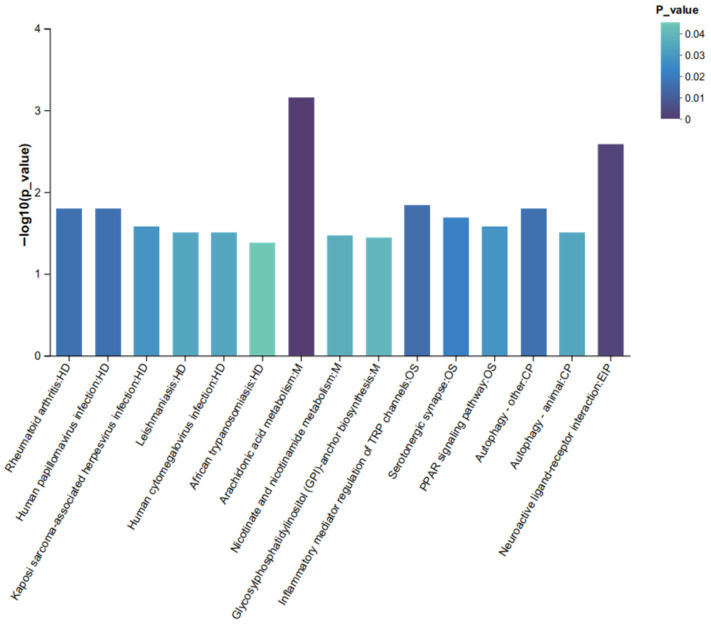
Metabolomic profiling and pathway enrichment analysis.

KEGG pathway enrichment analysis identified several pathways significantly enriched among differential metabolites (FDR < 0.05, Figure 10), including arachidonic acid metabolism, nicotinate and nicotinamide metabolism, the PPAR signaling pathway, and serotonergic synapse (5-hydroxytryptaminergic synapse). Although ‘tryptophan metabolism’ (ko00380) did not reach statistical significance in the global enrichment analysis, targeted examination of individual tryptophan-related metabolites revealed significant alterations.

The bubble plot shows significantly enriched KEGG pathways among differentially abundant metabolites (FDR < 0.05). The size of each bubble represents the number of metabolites annotated to the pathway; the color represents the pathway category: HD (human diseases), OS (organic systems), M (metabolism), and EIP (environmental information processing). The *x*-axis represents the enrichment ratio (rich factor), and the *y*-axis shows pathway names. Statistical analysis: Metabolite pathway enrichment was performed using Fisher’s exact test with Benjamini-Hochberg FDR correction (FDR < 0.05 considered significant). Metabolites were identified using OPLS-DA (VIP > 1.0, *p* < 0.05, FDR < 0.05). Sample size: *n* = 6 mice per group (solvent control: *n* = 6; PFAS-exposed: *n* = 6).

### 2.6. Integrated Analysis of Transcriptomics and Metabolomics

#### 2.6.1. Expression Correlation Analysis

**Figure 11:**
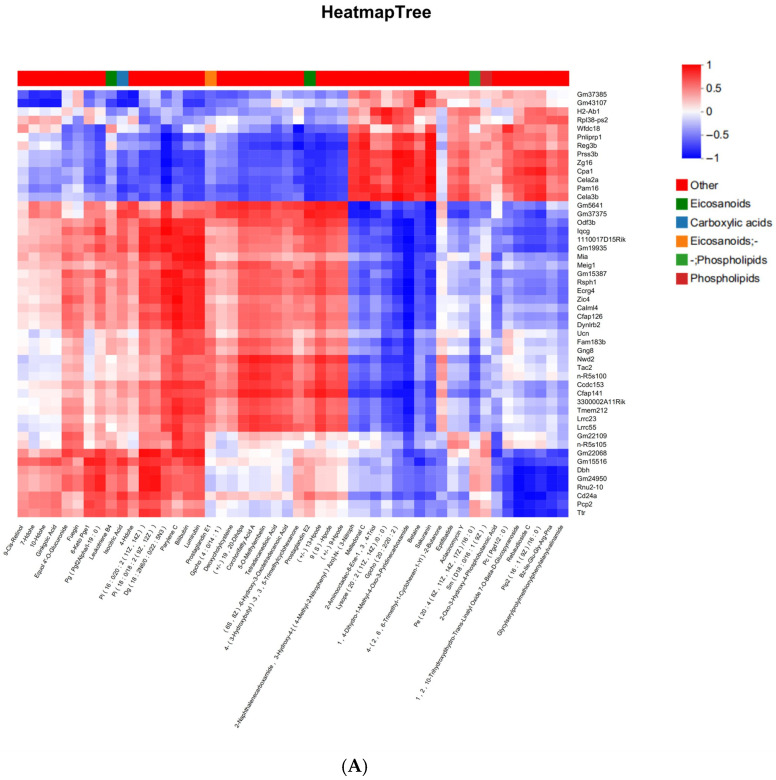

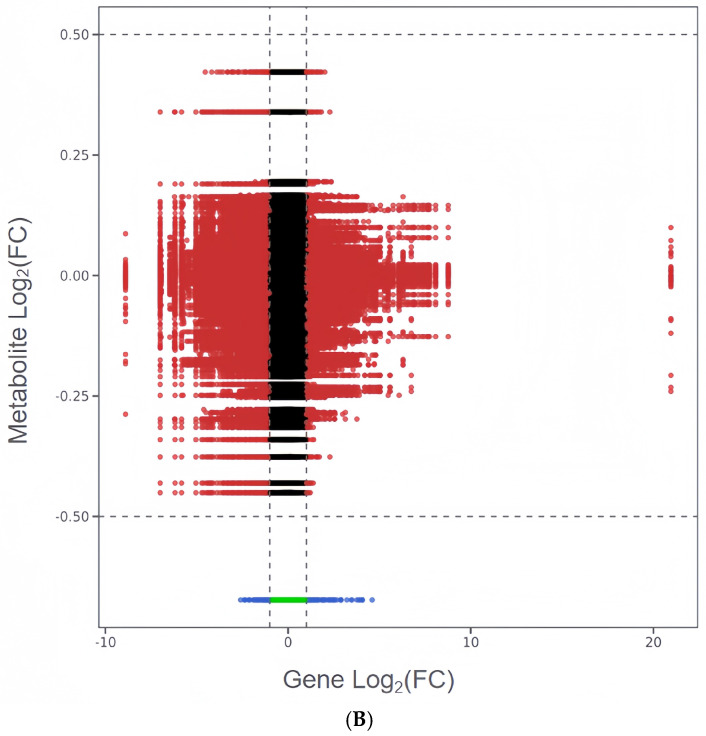
(**A**) Spearman correlation heatmap of differentially expressed genes (DEGs) and differentially abundant metabolites (DAMs) in mouse midbrain following PFAS mixture exposure. Rows: Differentially expressed genes (*n* = 315). For visual clarity, only genes with |Spearman r| > 0.6 are shown. Columns: Differentially abundant metabolites (*n* = 130). For visual clarity, only metabolites with |Spearman r| > 0.6 are shown. Color scale: Spearman correlation coefficient (r). Red indicates positive correlation (r > 0); blue indicates negative correlation (r < 0). Color intensity reflects the absolute correlation strength (darker = stronger |r|). Statistical significance: Only correlations with FDR < 0.05 (Benjamini–Hochberg corrected) are displayed. Highlighted pair: The red box indicates the strong positive correlation between *Dbh* expression and PI(16:0/20:2(11Z,14Z)) abundance (r = 0.903, FDR = 0.012). Data were derived from *n* = 3 mice per group (transcriptomics) and *n* = 6 mice per group (metabolomics). Color bar with range −1 to +1. (**B**) Nine-quadrant plot of gene–metabolite correlations. Each point represents a gene–metabolite pair. Quadrant boundaries are defined by black dashed lines based on log2(fold change) thresholds (|log_2_FC| ≥ 1.0) and significance (FDR < 0.05). Quadrant 7 (bottom-left, highlighted with light red background): Upregulated genes (log_2_FC > 0) with downregulated metabolites (log_2_FC < 0) showing positive correlation (Spearman r > 0.8). Light red background or light red box: Represents upregulated genes + downregulated metabolites + positive correlation (r > 0.8). Represents a special molecular pair with “inverse changes but highly positive correlation,” including the example pair *Dbh*-PI(16:0/20:2(11Z,14Z)). This quadrant contains the *Dbh*-PI(16:0/20:2(11Z,14Z)) pair (r = 0.903). Quadrant 9 (bottom-right): Both genes and metabolites downregulated (coordinated downregulation, r > 0.8). Quadrant 1 (top-left): Both genes and metabolites upregulated (coordinated upregulation, r > 0.8). Quadrants 3 and 7: Inverse relationships (gene down/metabolite up, or gene up/metabolite down). Quadrant 5 (center): Non-significant molecular changes (|log_2_FC| < 1, FDR > 0.05).

Integrative Spearman correlation analysis between DEGs and DAMs was visualized as a correlation heatmap (Figure 11A). In this heatmap, rows represent individual DEGs, columns represent individual DAMs, and color intensity reflects the Spearman correlation coefficient (red: positive correlation; blue: negative correlation). This analysis revealed a strong positive correlation between *Dbh* expression and the phosphatidylinositol species PI(16:0/20:2(11Z,14Z)) (r = 0.903, FDR = 0.012), as highlighted in Figure 11A. Significant molecular covariations were identified, including a strong positive correlation between *Dbh* expression and phosphatidylinositol PI(16:0/20:2(11Z,14Z)) abundance (r = 0.903) and and an inverse correlation between *Cfap126* and the pyridine derivative 1-methyl-4-oxo-1,4-dihydropyridine-3-carboxamide (r = −0.976). A nine-quadrant correlation matrix was generated to visualize high-confidence molecular relationships (Figure 11B). Quadrant boundaries were defined using thresholds of |log_2_FC| ≥ 1.0 and FDR < 0.05 for significant changes (Benjamini-Hochberg correction). The nine-quadrant plot revealed distinct co-regulation patterns: Quadrant 7 (bottom-left, highlighted): Characterized by upregulated genes and downregulated metabolites exhibiting a positive correlation (r > 0.8). This quadrant included the PI(16:0/20:2)-*Dbh* pair (r = 0.903), suggesting an inverse relationship between this lipid species and *Dbh* expression. Quadrant 9 (bottom-right): Genes and metabolites both downregulated (coordinated downregulation, r > 0.8). Quadrant 1 (top-left): Genes and metabolites both upregulated (coordinated upregulation, r > 0.8). Quadrants 3 and 7: Inverse relationships (gene down/metabolite up, or gene up/metabolite down) Quadrant 5: Non-significant molecular changes (|log_2_FC| < 1, FDR > 0.05); Quadrant 4 (middle-left): Discordant changes with elevated metabolite abundance but unaltered or downregulated gene expression (r < −0.8); Quadrants 6, 8, 9: Inverse associations featuring gene upregulation coupled with unaltered or reduced metabolite levels (r < −0.8).

#### 2.6.2. KEGG Pathway Annotation Analysis

**Figure 12:**
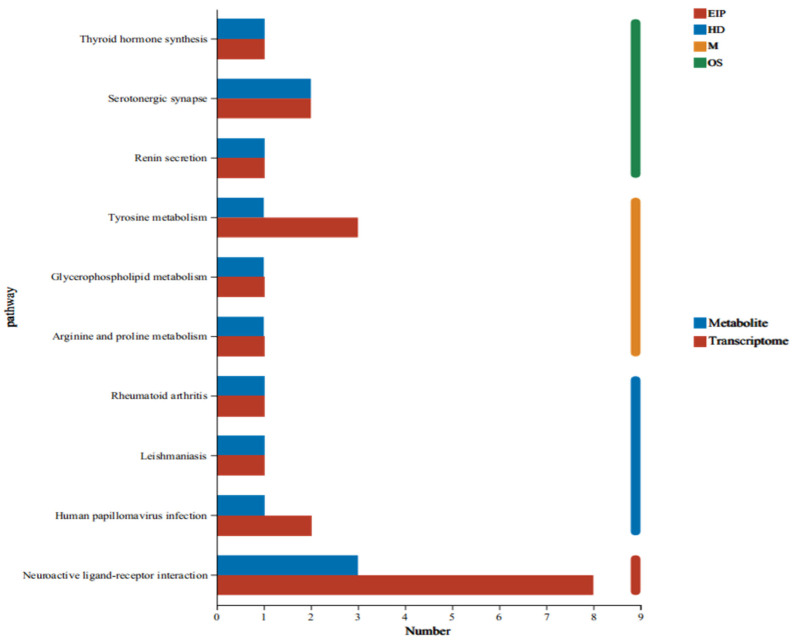
The number of DEGs and DMs involved in functional pathways.

To elucidate functional crosstalk between DEGs and differentially abundant metabolites (DAMs), we performed integrated pathway enrichment analysis by mapping multi-omics features to KEGG pathways using topology-aware annotation (Figure 12). The results indicated that DEGs and DAMs induced by PFAS were mainly involved in neuroactive ligand-receptor interactions, papillomavirus infection, leishmaniasis, rheumatoid arthritis, arginine and proline metabolism, the citrate cycle (TCA cycle), glycerophospholipid metabolism, TM, renin secretion, 5-HT, and thyroid hormone anabolism.

The vertical coordinate shows the KEGG pathway name; the horizontal coordinate shows the number of DEGs (red bars) or DAMs (blue bars) annotated to each pathway. The color band on the right represents the first-level KEGG classification: EIP (environmental information processing), HD (human diseases), M (metabolism), and OS (organic systems). Statistical analysis: Pathways were selected based on overlap with at least one DEG (FDR < 0.05, |log_2_FC| ≥ 1.5) or DAM (VIP > 1.0, *p* < 0.05, FDR < 0.05). Enrichment analysis used a hypergeometric test with Benjamini-Hochberg correction. Sample size: Transcriptomics: *n* = 3 mice per group; Metabolomics: *n* = 6 mice per group.

**Figure 13:**
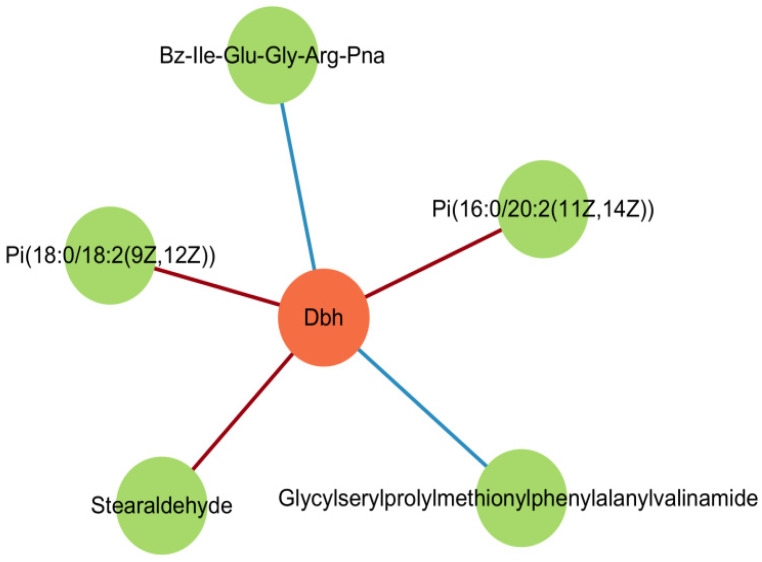
Differentially expressed genes and related differences in metabolite reaction networks (*n*= 3) note: color of genes; Green represents metabolites. Node colors: Green nodes represent metabolites; colored nodes represent genes (colors correspond to molecular function categories). Edge color: Red edges indicate positive correlation; blue edges indicate negative correlation. Edge thickness represents the absolute Spearman correlation coefficient (thicker = stronger correlation). Only correlations with |Spearman r| > 0.8 and FDR < 0.05 are shown. Highlighted pair: The strong positive correlation between *Dbh* expression and PI(16:0/20:2(11Z,14Z)) abundance is shown (r = 0.903, FDR = 0.012). Statistical analysis: Spearman correlation analysis was performed between all DEGs and DAMs, with Benjamini-Hochberg FDR correction (FDR < 0.05 considered significant). Network visualization was performed using Cytoscape (v3.9.1). Sample size: Transcriptomics: *n* = 3 mice per group; Metabolomics: *n* = 6 mice per group.

Within the TM/5-HT signaling pathway, an integrated analysis of transcriptomic-metabolomic correlations revealed significant associations between DEGs and DAMs. Specifically, *Dbh* expression demonstrated strong positive covariation with: Phosphatidylinositol species: PI(16:0/20:2(11Z,14Z)) (r = 0.903) and PI(18:0/18:2(9Z,12Z)) (r = 0.861); Aldehyde metabolite: Octadecanal (stearaldehyde; r = 0.842); Peptidic metabolites: Bz-Ile-Glu-Gly-Arg-pNA (r = 0.827) and Gly-Ser-Pro-Met-Phe-Val-NH_2_ (r = 0.809, FDR = 0.029). Notably, PI(16:0/20:2(11Z,14Z)) exhibited the strongest correlation with *Dbh* expression (r = 0.903) among all metabolite-gene pairs. This strong correlation identifies PI(16:0/20:2) as a candidate molecular marker for future hypothesis-driven studies. However, correlation does not imply causation, and functional studies are required to determine whether PI(16:0/20:2) plays a direct role in *Dbh* regulation or dopamine metabolism (Figure 13). These findings suggest that TM/5-HT-related annotations co-occur with lipid alterations in our dataset.

### 2.7. Mixed Exposure to PFASs Perturbates Intestinal Microbiota Homeostasis in Mice

#### 2.7.1. Analysis of Intestinal Microbiota Diversity

**Figure 14:**
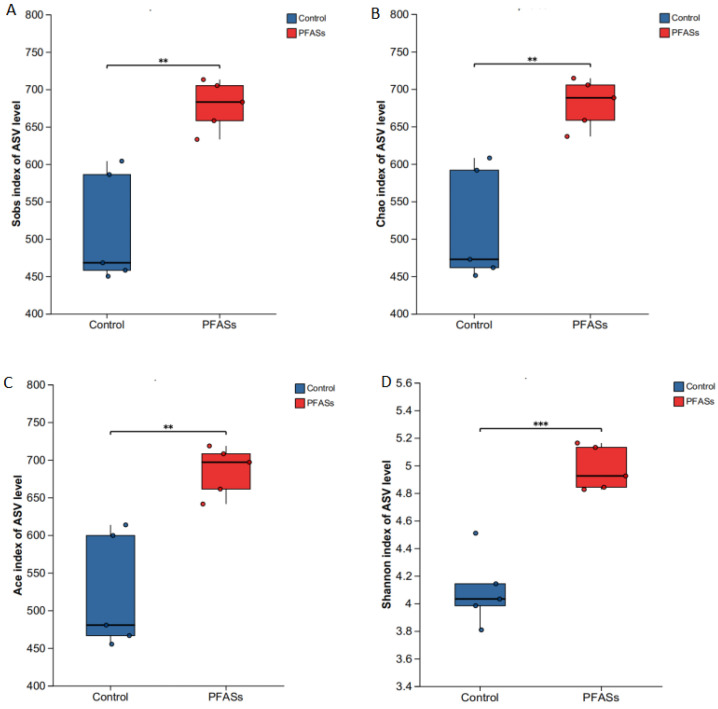
Analysis of colonic microbiota diversity. Violin plots show four alpha diversity metrics: (**A**) Observed species (S_obs_), (**B**) Chao1 richness estimator, (**C**) ACE index, and (**D**) Shannon diversity index. Each point represents an individual mouse. Boxes indicate the interquartile range (IQR); whiskers represent 1.5 × IQR; horizontal lines indicate medians. Statistical analysis: Unpaired two-tailed Student’s *t*-test was used after confirming normality (Shapiro-Wilk test) and homogeneity of variance (Levene’s test). Benjamini-Hochberg FDR correction was applied for multiple comparisons (FDR < 0.05 considered significant). “**” indicates *p* < 0.01 and “***” indicates *p* < 0.001 compared with solvent control group. Sample size: *n* = 6 mice per group (solvent control: *n* = 6; PFAS-exposed: *n* = 6).

Alpha diversity metrics quantify microbial community complexity at the individual sample level. Our analysis comprehensively assessed community richness (reflected by observed species [S_obs_], Chao1, and ACE indices) and heterogeneity (Shannon diversity index) across experimental groups (Figure 14). Crucially, combined PFAS exposure elicited significant elevation in all α-diversity indices relative to vehicle controls (S_obs_: 48% increase; Chao1: 39% increase; ACE: 42% increase; Shannon: 1.8-fold elevation; *p* < 0.01). This cohort-wide enhancement demonstrates that chemical exposure remodels gut ecosystem architecture by amplifying both taxonomic richness and phylogenetic heterogeneity.

Beta diversity was assessed using principal coordinate analysis (PCoA) based on Bray-Curtis dissimilarity to visualize structural differences in gut microbial communities (Supporting Information Figure S9). While the PFAS-exposed and control groups showed partial separation along the primary coordinate axes, overlap was observed (one control sample clustered within the PFAS group; one PFAS sample clustered within the control group). Statistical confirmation using analysis of similarity (ANOSIM) revealed a significant but modest difference between groups (R = 0.533, *p* = 0.016), indicating that PFAS exposure explains a portion, but not all, of the variance in gut microbial community structure.

#### 2.7.2. Community Composition Analysis of Intestinal Microbiota

**Figure 15:**
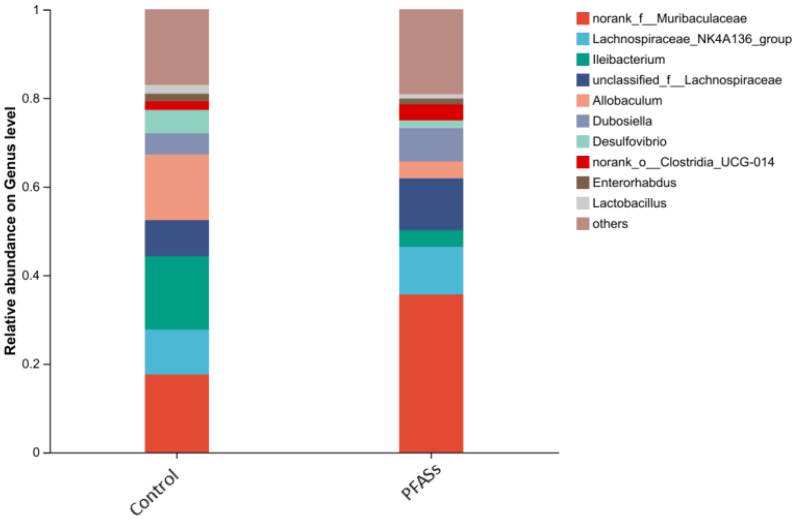
Community composition Bar diagram at the phylum level.

Phylum-level taxonomic profiling revealed the ten highest-abundance intestinal microbiota, comprising Firmicutes, Bacteroidota, Desulfobacterota, Actinobacteriota, Patescibacteria, Campilobacterota, Proteobacteria, Deferribacterota, Cyanobacteria, and Verrucomicrobiota (Figure 15). Strikingly, compositional shifts were observed between cohorts: Firmicutes predominated in controls (relative abundance: 62.7 ± 5.2%), while Bacteroidota emerged as the dominant phylum in PFAS-exposed specimens (51.3 ± 4.8%). This marked inversion of dominant taxa indicates significant chemical-induced restructuring of the intestinal ecosystem.

**Figure 16:**
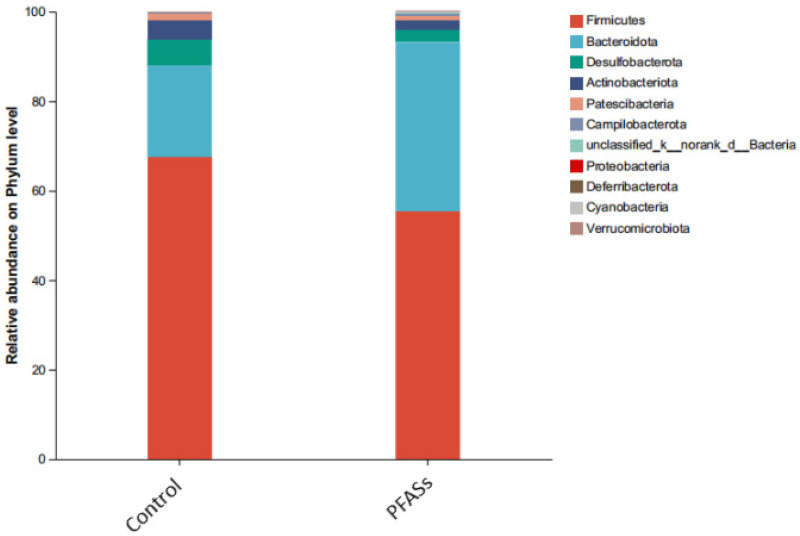
Community composition Bar diagram at the genus level.

Genus-level analysis identified the ten predominant bacterial taxa ranked by relative abundance: unclassified *Muribaculaceae*, *Lachnospiraceae* NK4A136_group, *Ileibacterium*, unclassified *Lachnospiraceae*, *Allobaculum*, *Dubosiella*, *Desulfovibrio*, *Clostridium* UCG-014, *Enterorhabdus*, and *Lactobacillus* (Figure 16).

**Figure 17:**
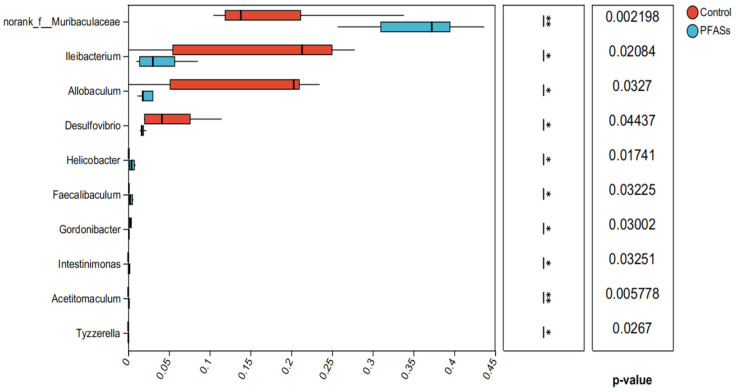
Box-type diagram of species difference analysis at the genus level. “*” indicates *p* < 0.05, “**” indicates *p* < 0.01.

Intergroup differences in microbial abundance were assessed using unpaired two-tailed Student’s *t*-tests. As depicted in Figure 17, PFAS exposure significantly reduced the relative abundance of three key taxa: *Ileibacteriaceae* (1.8-fold decrease, t = 4.32, *p* = 0.003), *Heterorhabdus* (2.1-fold reduction, t = 5.07, *p* = 0.001), and *Desulfovibrio* (3.2-fold decline, t = 6.84, *p* < 0.001) compared to controls.

## 3. Discussion

This investigation identifies transcriptomic and metabolomic correlates that suggest a potential role for TM/5-HT pathway disruption in PFAS mixture neurotoxicity. However, given the correlative nature of multi-omics data, these findings should be interpreted as hypothesis-generating rather than mechanistic proof. Compared with extant literature, our study advances three pivotal insights: Pathological Validation: Environmentally relevant PFAS mixtures recapitulate cardinal Parkinsonian pathology, including increased α-syn expression and reduced TH expression. Metabolic Hub Identification: Phosphatidylinositol PI(16:0/20:2(11Z,14Z)) serves as a critical signaling node orchestrating TM/5-HT pathway crosstalk. 3. Novel Pathogenic Cascade: Our integrated analysis revealed correlative changes between neurotransmitter-related genes (e.g., *Dbh*, *Tyrp1*, *Htr5b*) and calcium-linked lipid metabolites (e.g., PI(16:0/20:2)). Specifically, *Dbh* expression showed a strong positive correlation with PI(16:0/20:2) abundance (r = 0.903). While these correlations raise the hypothesis that PFAS exposure may affect dopaminergic neurons through interconnected neurotransmitter and calcium signaling pathways, direct functional evidence is lacking. We therefore present this as a hypothesis-generating observation rather than a validated mechanism.

Mice in the PFAS group exhibited typical Parkinson-like phenotypes, including spatial memory deficits (prolonged water maze avoidance latency) and locomotor impairment (increased pole-climbing T-turn time) [24,25]. These findings diverge markedly from reported PFOS monotherapy effects [26], demonstrating significantly exacerbated motor dysfunction in PFAS mixture-exposed cohorts, indicative of a synergistic neurotoxic interaction. Notably, PFAS exposure significantly reduced total locomotor activity in the open field without concomitant anxiety-like behaviors. This phenotype distinctively contrasts with the MPTP neurotoxin model [25] (which exhibits both hypokinesia and increased anxiety), suggesting selective targeting of cortico-basal ganglia-thalamo-cortical motor circuits by PFAS mixtures.

Neuronal degeneration represents a characteristic neuropathological hallmark in both chronic neurodegenerative disorders and acute neurotraumatic conditions [27], serving as a quantifiable index of neurological compromise. Midbrain dopaminergic neurodegeneration manifests through three cardinal pathomechanisms in the substantia nigra pars compacta: Selective neuronal loss: reduction in tyrosine hydroxylase-positive neurons; Mitochondrial bioenergetics failure, involving Complex I activity impairment and ATP synthesis deficits; Redox homeostasis collapse, marked by ROS overproduction and compromised antioxidant responses; α-Synuclein protofibril accumulation. We observed concomitant upregulation of α-synuclein expression and downregulation of TH expression in PFAS-exposed midbrains (Figure 6). While these correlative changes are consistent with patterns observed in early Parkinsonian pathology, we did not directly assess α-synuclein aggregation or oligomerization. Therefore, our data support increased α-synuclein expression but do not demonstrate the formation of pathogenic oligomers. This discovery extends the theoretical framework established by Drolet et al. [28]: Inhibition of TH catalytic activity profoundly compromises dopamine biosynthesis kinetics, resulting in reduction in striatal dopamine synthesis; critically, PFAS exposure induced significant synaptic dysfunction without concomitant neuronal loss. This dissociation between structural integrity and functional impairment suggests preferential targeting of synaptic machinery over direct neurotoxicity, providing empirical evidence for environmentally triggered subclinical neurodegeneration as defined by Jack et al. [29].

Transcriptomic profiling revealed dopamine β-hydroxylase (*Dbh*) as the most markedly downregulated gene. The downregulation of *Dbh* expression observed in our study is consistent with a scenario of reduced norepinephrine synthesis. In other model systems, *Dbh* inhibition has been shown to trigger feedback mechanisms that can suppress TH expression. Whether such a mechanism operates in our PFAS exposure model remains to be tested. This catecholaminergic disruption is mechanistically linked to prefrontal cortex-dependent cognitive impairment and motor dysfunction as demonstrated in translational models [30,31]. Tyrosinase-related protein 1 functions as a critical metalloenzyme primarily involved in the oxidative catalysis of tyrosine-derived intermediates during melanogenic biosynthesis within melanosomes [32,33]. In contrast, *Got1l1* downregulation perturbs anaplerotic flux by impairing the aspartate-glutamate transamination cycle, depleting oxaloacetate pools and compromising tricarboxylic acid cycle integrity [33]. Pathological perturbations along the phenylalanine-tyrosine metabolic axis generate a biosynthetic bottleneck for dopamine precursor availability, which is mechanistically linked to impaired motor performance and executive function deficits in translational models [34,35,36]. Critically, downregulation of serotonin receptors disrupts G protein-coupled receptor (GPCR)-mediated 5-HT signaling cascades [37,38]. This perturbation dysregulates the nucleus tractus solitarius (NTS)-mesocortical limbic loop, while L-DOPA intervention models confirm impaired dopaminergic tone regulation [39,40]. Collectively, these cross-pathway interactions establish a novel neurotoxicological paradigm that explains the supra-additive effects of PFAS mixtures versus individual compounds. This model both: Reveals synergistic neurotoxicity mechanisms underlying combined pollutant exposure; Identifies actionable molecular targets for environmentally triggered neurodegeneration pathologies [34,35].

Research has revealed that changes in *Muribaculaceae* abundance correlate with systemic inflammatory responses. In mouse models chronically exposed to microcystin-LR (MC-LR), increased *Muribaculaceae* promote systemic inflammation via the ornithine metabolism pathway, while neuroinflammation is a known precipitating factor for dopaminergic neuron damage. In an alcohol withdrawal-induced depressive rat model, reduced *Muribaculaceae* abundance correlated with depressive-like behaviors, while lavender essential oil intervention improved behavioral performance by increasing its abundance [41]. Although this study did not directly examine dopaminergic neurons, depressive behaviors are closely linked to dopaminergic system function. *Muribaculaceae* participate in short-chain fatty acid (SCFA) and amino acid metabolism [42]. SCFAs regulate neuroinflammation via the blood-brain barrier, while ornithine metabolism influences neurotransmitter balance and may indirectly affect dopaminergic neurons.

Integrated transcriptomic-metabolomic analyses revealed that PFAS converge on shared pathophysiological axes through: The tyrosine metabolic axis perturbation manifested as: Catalytic Dysregulation, *Dbh* suppression, and *Got1l1* downregulation; Pathway Flux Impairment, Phenylalanine-tyrosine metabolic bottleneck, and Anaplerotic substrate depletion. Mitochondrial Bioenergetic Crisis; TCA cycle flux reduction, Compromised acetyl-CoA shuttling.

This dual enzymatic deficiency establishes a metabolic gridlock at the tyrosine-TCA interface, quantified by flux control coefficients. Environmental xenobiotics, particularly PFAS, are documented to disrupt monoamine neurotransmitter biosynthesis through targeted impairment of rate-limiting synthases [43]. Our multi-omics profiling further establishes the TM axis as a vulnerable hub for PFAS-induced neurotoxicity, evidenced by: Pathway-specific transcriptional suppression, Enzyme kinetic compromise, and metabolite pool depletion. The 5-HT signaling axis perturbation involves transcriptional suppression of *Htr5b* and *Gng8*, impairing G_αi/o_-coupled GPCR signal transduction. This molecular deficit mechanistically aligns with Luchicchi et al.’s demonstration of 5-HT receptor crosstalk via allosteric modulation [40], establishing conserved GPCR network vulnerability. Within the lipid homeostasis network, phosphatidylinositol 16:0/20:2 (PI(16:0/20:2)) depletion exhibited a strong positive correlation with *Dbh* transcriptional suppression. This phospholipid deficit potentiates calcium dysregulation via impaired PIP_2_ hydrolysis, evidenced by: PLCγ signaling attenuation, IP receptor dysfunction, and CaMKII autophosphorylation deficit.

This study conducted a multidimensional systematic assessment of the neurotoxic effects elicited by mixed PFAS exposure on midbrain dopaminergic neurons. Utilizing environmentally relevant concentrations to simulate real-world scenarios, we recapitulated Parkinson’s disease-like motor deficits in behavioral assays. TEM revealed early-stage ultrastructural pathologies, including mitochondrial swelling and neuronal degeneration. Crucially, to our knowledge, this is the first report in a PFAS exposure model showing correlative changes between α-synuclein upregulation and TH downregulation. However, causality between these events remains to be established through functional studies (e.g., α-syn knockdown or overexpression experiments). Furthermore, integrative multi-omics analysis uncovered core pathogenic mechanisms involving the concurrent disruption of the TM/5-HT pathway and dysregulation of calcium signaling. Based on these findings, we propose a novel “energy metabolism-synaptic plasticity” dual-injury hypothesis supported by our data. This work not only identifies novel candidate molecular correlates for PFAS neurotoxicity but also provides a mechanistic foundation for developing intervention strategies. The experimental design employing environmentally relevant doses, a complex mixture model, and in-depth mechanistic exploration significantly enhances the scientific rigor and translational potential of the findings.

However, this investigation is constrained by three principal limitations: Temporal resolution deficit in quantifying progressive neurodegeneration during chronic exposure; Unvalidated causal necessity of hub targets; Simplified exposure modeling failing to recapitulate human co-exposure complexity (for example, PFAS-heavy metal mixtures). Future research priorities should emphasize: Development of neuroprotectants targeting TM/5-HT pathway convergence nodes; Mechanistic dissection of cumulative neurotoxicity from chemical mixtures; Multi-tiered validation encompassing: Large-scale exposome-wide association studies; In-human trials of pathway-targeted diagnostics.

Several limitations should be considered. First, all multi-omics analyses (transcriptomics, metabolomics, and 16S rRNA profiling) yielded correlative data. The proposed TM/5-HT pathway cascade, highlighting the central role of PI(16:0/20:2) and hub genes such as Dnali1, lacks functional validation (e.g., gene editing, pharmacological intervention, or calcium imaging). Second, behavioral changes described as “parkinsonism-related” do not fully recapitulate human Parkinson’s disease, as we observed no progressive TH+ neuron loss or resting tremor. Third, our mixture model included only three short-chain PFAS without co-exposure to heavy metals or other pollutants commonly found in human environments. Fourth, only male mice were used. Therefore, causal inference and translational extrapolation require further experimental support.

Several conceptual limitations should be emphasized. First, our study was designed as an exploratory multi-omics analysis, not as a mechanistic validation study. Therefore, all proposed pathways (including TM/5-HT) and hub genes remain correlative candidates requiring functional testing. Second, the inclusion of gut microbiota analysis was exploratory; we do not claim causal evidence for a gut-brain axis mechanism. Third, our findings are derived from a male mouse model with a simplified three-compound PFAS mixture and a 28-day exposure period. Direct extrapolation to human neurodegenerative diseases (e.g., Parkinson’s disease) is not warranted based on these data alone. Fourth, we have not validated any therapeutic target; the term “target” in the original manuscript was used prematurely and has been removed.

A critical limitation of this study is the discrepancy between the mg/kg doses used and real-world environmental exposure levels (typically in the μg/kg range). The dosing regimen (37.5 mg/kg PFBS, 15 mg/kg PFBA, 1 mg/kg PFHxA) was designed to induce detectable neurobehavioral and multi-omics alterations within a 28-day exposure window, which required doses substantially higher than those found in contaminated water sources. While we anchored our dose selection to environmental monitoring data (Daling River basin) and applied a 1000-fold safety factor consistent with exploratory toxicology practice, the final doses do not represent environmentally relevant concentrations. The doses used in this study (37.5 mg/kg) were selected based on the higher metabolic rate of mice compared to humans. According to the Human Equivalent Dose (HED) calculation, this dose aims to mimic the cumulative effects of long-term environmental exposure in a sub-chronic laboratory setting. Future studies should focus on even lower doses to better simulate real-world human exposure levels.

## 4. Materials and Methods

### 4.1. Chemical Dosing and Rationale

Environmental exposure basis. PFAS concentrations in surface water were obtained from a basin-scale assessment of the Daling River, an industrialized region in northern China [44]: PFBS (2.90 μg/L), PFBA (1.35 μg/L), and PFHxA (0.06 μg/L). These three short-chain PFAS were selected because they represent the predominant congeners detected in both environmental samples and brain tissues in previous biomonitoring studies [21].

Dose selection rationale. The doses used in this study were not intended to directly mimic environmental levels, but rather to induce detectable neurobehavioral and multi-omics alterations within a 28-day exposure window for exploratory toxicology purposes. The final doses were determined based on three considerations:

Pilot study results. Preliminary dose-finding experiments using PFBS alone at 5, 10, 20, and 40 mg/kg/day for 28 days showed that doses below 10 mg/kg produced no significant changes in open field activity or midbrain TH expression. A dose of 37.5 mg/kg PFBS was selected as the highest dose that did not cause overt toxicity (e.g., weight loss, lethargy, mortality) based on the established 90-day oral NOAEL for PFBS in mice (50 mg/kg/day).

Equipotent mixture design. Based on published relative potency estimates for neurotoxicity (PFBA:PFOA ratio from Liu et al. 2023 [17]; PFHxA:PFOS ratio from Ivantsova et al. 2024 [10]), PFBA and PFHxA doses were scaled to maintain approximate equipotency with PFBS: PFBA at 15 mg/kg/day (0.4× PFBS dose) and PFHxA at 1 mg/kg/day (0.027× PFBS dose).

Consistency with literature. The final mixture (37.5 mg/kg PFBS + 15 mg/kg PFBA + 1 mg/kg PFHxA) falls within the range of PFAS doses used in subchronic mouse neurotoxicity studies (typically 10–50 mg/kg for individual compounds) [3,21,26].

Dosing protocol. Test solutions were freshly prepared in a vehicle containing 5% DMSO (*v*/*v*) in sterile pyrogen-free water and administered via oral gavage at 10 mL/kg body weight daily for 28 consecutive days. The vehicle control group received an equal volume of 5% DMSO in saline. All solutions were used within 2 h of preparation.

### 4.2. Animal Handling and Sample Collection

Thirty specific-pathogen-free (SPF) male C57BL/6J mice (age: 8–10 weeks) were obtained from Jiangsu Huachuang Xinnuo Co., Ltd. (Taizhou, China; SCXK 2023-0012). Following random allocation, mice were randomly assigned to two groups: (1) vehicle control (5% DMSO in saline) and (2) PFAS mixture-exposed (37.5 mg/kg/day PFBS + 15 mg/kg/day PFBA + 1 mg/kg/day PFHxA). All mice were housed under standardized conditions (22 ± 1 °C, 55 ± 5% humidity, 12:12 light-dark cycle) at the AAALAC-accredited Experimental Animal Center of Guilin Medical University. Following a 7-day acclimation period with standard rodent chow ad libitum under controlled conditions (temperature: 23 ± 2 °C; light cycle: 12/12-h light/dark), test substances were administered via oral gavage at 10 mL/kg body weight daily for 28 consecutive days. Upon completion of the 28-day exposure regimen, mice underwent comprehensive behavioral phenotyping, including: (i) the open field assay for locomotor activity, (ii) the Morris water maze for spatial memory, (iii) the pole climbing test for motor coordination, and (iv) automated gait analysis. Following behavioral assessments, animals were euthanized via cervical dislocation under deep anesthesia. Midbrain tissue was immediately dissected on a chilled platform (4 °C), snap-frozen in liquid nitrogen, and stored at −80 °C for subsequent neurochemical analyses. All animal experiments in this study were designed, conducted, analyzed, and reported in strict accordance with the Animal Research: Reporting of In Vivo Experiments (ARRIVE) Guidelines 2.0. Experimental design and blinding: A total of 30 male C57BL/6J mice (8–10 weeks old) were randomly assigned to two groups (*n* = 15 per group) using a random number generator: (1) vehicle control (5% DMSO in saline) and (2) PFAS mixture (37.5 mg/kg PFBS + 15 mg/kg PFBA + 1 mg/kg PFHxA). Sample size was determined based on power analysis (assuming effect size d = 1.2, α = 0.05, power = 0.8). Investigators performing behavioral tests, histological analyses, and image quantification were blinded to group allocation. Blinding codes were broken only after data collection was complete.

### 4.3. Behavioral Science

#### 4.3.1. Morris Water Maze (MWM) Test

The Morris Water Maze is commonly used for investigating spatial memory and learning abilities in mice. It consists of a circular basin with a height of 50 cm and a diameter of 180 cm, which contained at least 24 cm of water at 25 °C, four equal quadrants, and a transparent circular platform that is submerged 1.0 cm below the surface of the water, there are obvious circular images hanging on the wall. Before the probe test, all mice were scheduled for initial training. The mice were allowed to stay on the platform for 1 min to learn the location of the hidden platform for escaping and memorize the spatial location of this platform. And then, mice were placed into the water facing the middle of the wall, which is one of the four quadrants, and allowed to look for and climb upon the platform. Mice that failed to locate the platform within 1 min were gently guided to it. All mice were subjected to four trials with four different starting positions in one day. The interval for each mouse per trial was at least 15 min, and training lasted 4 days.

On the fifth day, the probe test was implemented. We used the Any-Maze animal behavior analysis system, provided by Stoelting (Wood Dale, IL, USA), to track and record the movement of experimental animals. The transparent circular platform was removed, and the testing mice were placed into the quadrant opposite to the place where the platform had been hidden. We recorded escape latency (defined as the time from placing the mouse in the basin until it first found the platform), the number of crossings over the hidden platform location, and swimming speed within 1 min.

#### 4.3.2. Open Field Test (OFT)

The open field test (OFT) was employed to evaluate spontaneous locomotor activity and anxiety-like behavior. Testing occurred in a square PVC arena (1 m × 1 m × 0.4 m) with white interior surfaces, divided into 16 equal quadrants (0.25 m × 0.25 m) by virtual gridlines. Each mouse was gently placed in the central quadrant and allowed to explore freely for 5 min under 300 lux of illumination. Locomotor parameters (total distance traveled, center duration, and mean velocity) were automatically tracked and recorded using ANY-maze™ software (v6.3, Stoelting Co., Wood Dale, IL, USA). The arena was thoroughly cleaned with 70% ethanol between trials to eliminate olfactory cues.

#### 4.3.3. Pole Test (PT)

The pole climbing test was employed to evaluate neuromotor coordination, limb strength, and vestibulomotor function in rodents, consistent with protocols for neurotoxicity assessment (OECD TG 424). The apparatus featured a vertically oriented cylindrical pole (diameter: 10 mm; height: 50 cm) affixed to a weighted base, surmounted by a spherical platform (Ø25 mm). To optimize surface traction, the pole was wrapped with textured adhesive tape, while 10 cm deep corncob bedding provided impact protection. After two consecutive days of habituation allowing unrestricted exploration, each mouse was positioned in a head-down orientation at the apex and permitted to descend freely. Descent latency was digitally recorded from snout contact with the bedding surface, with three trials performed at 15-min intervals.

#### 4.3.4. Gait Analysis Experiment

Gait analysis quantitatively evaluates locomotor symmetry, interlimb coordination, and neuromotor dysfunction through spatiotemporal parameter measurement. For the assessment, mice were placed in a standardized runway apparatus (0.5 m × 0.08 m × 0.15 m) lined with pressure-sensitive paper, terminating in a goal box containing standard chow. Paw prints were collected during voluntary ambulation toward the food reward, with subsequent analysis of stride length, base width, and step-sequence patterns. Animals were motivated to traverse the runway toward a food reward located at the distal end. To enable gait recording, the apparatus floor was lined with chromatographic paper (0.3 m × 0.08 m; Whatman Grade 1, Zhuoyi, Guilin, China) precisely sized to the runway dimensions. Dorsal surfaces of forepaws and hindpaws were coated with non-toxic, water-soluble dyes (red and blue chromatographic inks, respectively). Mice were gently positioned at the runway start point facing the goal box. Upon reaching the terminal end, animals were immediately removed and returned to their home cages. The gait recording substrate was then retrieved and air-dried in a controlled environment (22 ± 1 °C, 45–55% RH) prior to digitization.

### 4.4. Histopathological Analysis

Midbrain tissues were fixed by immersion in 4.0% (*w*/*v*) paraformaldehyde (PFA; pH 7.4) for 24 h at 4 °C. Following post-fixation washing in PBS, samples were dehydrated through a graded ethanol series (70–100%), cleared in xylene, and embedded in paraffin blocks. Serial coronal sections (5 μm thickness) were prepared using a rotary microtome (Leica RM2245, Leica Microsystems, Wetzlar, Germany) and stained with hematoxylin and eosin (H&E) according to Bancroft’s protocol. Histopathological alterations in midbrain tissues were evaluated by light microscopy (Zeiss Axio Imager M2, Oberkochen, Germany) using H&E-stained sections. Semi-quantitative assessment of neuronal atrophy was performed using the following criteria: 0 = no visible changes; 1 = mild (focal neuronal shrinkage, <25% of neurons affected); 2 = moderate (multifocal atrophy, 25–50% affected); 3 = severe (diffuse atrophy, >50% affected). All scoring was performed by an investigator blinded to group assignment.

### 4.5. Immunohistochemistry

Mouse midbrain tissues were fixed in 4% (*w*/*v*) paraformaldehyde for 48 h at 4 °C, dehydrated through a graded ethanol series (70–100%), and embedded in paraffin. Serial sections (4–5 μm thickness) were dewaxed in xylene, rehydrated through graded ethanols, and underwent heat-induced epitope retrieval (HIER) in 10 mM citrate buffer (pH 6.0) at 95 °C for 20 min. Endogenous peroxidase activity was blocked with 3% H_2_O_2_ for 15 min. After blocking with 5% BSA for 1 h, sections were incubated overnight at 4 °C with the primary antibody, followed by HRP-conjugated secondary antibody for 1 h at room temperature. Diaminobenzidine (DAB) chromogenic development was monitored microscopically. The protocol was completed by counterstaining with hematoxylin and mounting with a resin-based medium. Immunoreactivity was quantified using light microscopy (Zeiss Axio Imager M2, Carl Zeiss AG, Oberkochen, Germany) with automated image analysis (ImageJ v1.53).

### 4.6. Ultrastructural Analysis by Transmission Electron Microscopy (TEM)

Prior to tissue collection, culture dishes containing 2.5% glutaraldehyde fixative were prepared. Midbrain tissues dissected from mice were immediately placed into these dishes. Following brief trimming, the tissues were sectioned. Upon completion of sectioning, the tissue sections were transferred into microcentrifuge tubes containing fresh electron microscopy-grade glutaraldehyde fixative. Subsequent processing steps included post-fixation with osmium tetroxide, sequential dehydration through a graded ethanol series, epoxy resin infiltration and embedding, polymerization of the resin, ultramicrotomy (Leica, Vienna, Austria) to obtain ultrathin sections (70–90 nm), and post-staining with uranyl acetate and lead citrate. The prepared grids were then examined under a transmission electron microscope (TEM) (FEI, Hillsboro, OR, USA). Images were acquired and subsequently analyzed.

### 4.7. Transcriptomics Analysis

#### 4.7.1. RNA Extraction and Library Construction

Total RNA was isolated from mouse tissues using TRIzol reagent (Invitrogen, Thermo Fisher Scientific, Waltham, MA, USA) according to the manufacturer’s instructions. RNA integrity was assessed using an Agilent 4200 TapeStation system (Agilent Technologies, Santa Clara, CA, USA). To prepare sequencing libraries, ribosomal RNA (rRNA) was depleted from total RNA samples. Subsequently, stranded cDNA libraries were constructed and subjected to paired-end sequencing on an Illumina platform by Biomarker Technologies Co., Ltd. Purified mRNA underwent fragmentation via enzymatic or chemical cleavage, generating templates for first-strand cDNA synthesis by reverse transcriptase. Following second-strand cDNA synthesis, the resulting double-stranded cDNA was amplified by PCR. The amplified fragments were purified, and libraries were constructed with insert sizes ranging from 300 to 400 bp. Paired-end sequencing was then performed on an Illumina HiSeq™ platform.

#### 4.7.2. RNA Sequencing Data Analysis

RNA sequencing (RNA-seq) analysis was performed on midbrain tissues dissected from three randomly selected mice per group (experimental and control). Raw sequencing reads underwent quality control and preprocessing, including adapter trimming and low-quality base filtering, to generate clean reads. Transcript abundance (TPM) was calculated using RSEM for visualization only. Differential expression was performed using DESeq2 based on gene-level raw read counts. Differential gene expression analysis was performed using the DESeq2 package with the significance threshold set at an adjusted *p*-value (padj) < 0.05 and |log_2_ fold change (log_2_FC)| ≥ log_2_(1.5). Genes meeting these criteria were identified as differentially expressed genes (DEGs). Principal Component Analysis (PCoA) incorporating all quality control (QC) samples was performed to assess potential batch effects. Additionally, volcano plots were employed to visualize the DEGs characteristic of PFAS exposure. Gene Ontology (GO) and Kyoto Encyclopedia of Genes and Genomes (KEGG) pathway analyses were performed on DEGs to identify enriched biological functions, pathways, and modules. Quality control and intra-group consistency: Prior to differential expression analysis, sample quality was assessed using principal component analysis (PCoA) based on the top 500 most variable genes. PCoA revealed clear separation between PFAS-exposed and control groups, with tight clustering of biological replicates within each group (Figure S9 in the Supporting Information). Pairwise Pearson correlation coefficients between replicates exceeded 0.95 in all cases, and the mean coefficient of variation (CV) for gene expression across replicates was 0.12 ± 0.03, indicating high intra-group consistency. These metrics support the reliability of downstream differential expression analysis despite the modest sample size (*n* = 3 per group).

### 4.8. Metabolomics Analysis

Sample preparation. Midbrain tissue samples (*n* = 6 per group) were homogenized in ice-cold 80% methanol (containing internal standards: 2-chlorophenylalanine at 0.2 μg/mL for positive mode, heptadecanoic acid at 0.5 μg/mL for negative mode). The mixture was vortexed for 30 s, sonicated for 10 min on ice, and centrifuged at 12,000× *g* for 15 min at 4 °C. The supernatant was collected and dried under nitrogen gas. Dried extracts were reconstituted in 100 μL of 10% methanol and filtered through a 0.22-μm PTFE membrane prior to LC-MS analysis.

LC-MS acquisition. Metabolomic profiling was performed using an ultra-high-performance liquid chromatography (UHPLC) system (Waters Acquity I-Class, Waters Corporation, Milford, MI, USA) coupled to a Q-Exactive Orbitrap mass spectrometer (Thermo Fisher Scientific). Separation was achieved on a Waters HSS T3 column (Waters Corporation, Milford, MI, USA) (2.1 × 100 mm, 1.8 μm) at 40 °C. Mobile phase A: water with 0.1% formic acid; mobile phase B: acetonitrile with 0.1% formic acid. The gradient elution program was: 0–1 min, 5% B; 1–12 min, 5–95% B; 12–14 min, 95% B; 14–15 min, 95–5% B; 15–17 min, 5% B. Flow rate was 0.3 mL/min. Mass spectra were acquired in positive and negative ion modes (full scan range: 70–1050 *m*/*z*; resolution: 70,000).

Data preprocessing. Raw LC-MS data were processed using Progenesis QI software (v2.4, Nonlinear Dynamics). Feature detection, alignment, and deconvolution were performed with default parameters. Metabolites were putatively annotated by matching retention time, accurate mass (mass error < 5 ppm), and MS/MS fragmentation spectra against the Human Metabolome Database (HMDB, v4.0), MassBank, and an in-house library.

Normalization and transformation. To correct for technical variation, total ion current (TIC) normalization was applied to each sample. Additionally, internal standard normalization was performed using the spiked-in standards (2-chlorophenylalanine for positive mode, heptadecanoic acid for negative mode). Following normalization, metabolite abundances were log_2_-transformed to approximate normality. Normality was confirmed using the Shapiro–Wilk test (*p* > 0.05 for >90% of metabolites).

Missing value handling. Metabolites with >50% missing values in either group were excluded from further analysis (17% of initially detected features). For the remaining metabolites, missing values (5.6% of total data points) were imputed using K-nearest neighbor (KNN) imputation with k = 5 (using the impute package in R).

Statistical analysis. For univariate analysis, an unpaired two-tailed Student’s *t*-test was performed for each metabolite between PFAS-exposed and control groups. For multivariate analysis, orthogonal partial least squares discriminant analysis (OPLS-DA) was performed using the ropls package (v1.30.0) in R with 7-fold cross validation. Variable importance in projection (VIP) scores were calculated to rank metabolite contribution to group separation.

Differential metabolite selection. A metabolite was considered significantly differentially abundant if it met all three of the following criteria: VIP > 1.0 (from OPLS-DA), *p* < 0.05 (from Student’s *t*-test) and false discovery rate (FDR) < 0.05 (Benjamini–Hochberg correction for multiple testing across 1234 features).

### 4.9. Intestinal 16S rRNA Gene Sequencing and Microbiota Analysis

Sample collection and DNA extraction: Fresh intestinal contents from the cecum and colon were collected from mice immediately after euthanasia and stored at −80 °C. Total genomic DNA was extracted using the QIAamp PowerFecal Pro DNA Kit (Qiagen, Hilden, Germany) according to the manufacturer’s protocol. DNA concentration and purity were assessed using a NanoDrop 2000 spectrophotometer (Thermo Fisher Scientific, USA), and integrity was verified by 1% agarose gel electrophoresis.

16S rRNA gene amplification and sequencing: The V3-V4 hypervariable region of the bacterial 16S rRNA gene was amplified using barcoded primers 338F (5′-ACTCCTACGGGAGGCAGCA-3′) and 806R (5′-GGACTACHVGGGTWTCTAAT-3′). PCR amplifications were performed in triplicate using TransStart Fastpfu DNA Polymerase (TransGen Biotech, Beijing, China) under the following conditions: initial denaturation at 95 °C for 3 min, followed by 27 cycles of 95 °C for 30 s, 55 °C for 30 s, 72 °C for 45 s, and a final extension at 72 °C for 10 min. Negative controls (no template) and positive controls (mock bacterial community) were included in each run.

Library preparation and sequencing: Amplicons were pooled, purified using the AxyPrep DNA Gel Extraction Kit (Axygen, Union City, CA, USA), and quantified using the QuantiFluor™-ST fluorometric system (Promega, Madison, WI, USA). Sequencing libraries were prepared using the TruSeq^®^ DNA Sample Prep Kit (Illumina, San Diego, CA, USA) and sequenced on an Illumina NovaSeq 6000 platform (2 × 250 bp paired-end) at Biomarker Technologies Co., Ltd. (Beijing, China).

Bioinformatics processing: Raw sequencing reads were demultiplexed and quality-filtered using QIIME2 (v2023.2) with the following criteria: (i) truncation of reads at positions with an average quality score < Q20; (ii) removal of reads containing ambiguous bases or mismatches to primer sequences; (iii) merging of paired-end reads using Vsearch (v2.15.0) with a minimum overlap of 30 bp. High-quality sequences were clustered into amplicon sequence variants (ASVs) using the DADA2 pipeline (via QIIME2). Taxonomy was assigned against the SILVA 138.1 reference database (99% similarity threshold) using a naive Bayes classifier. A rarefaction depth of 30,000 reads per sample was applied to normalize sequencing depth based on rarefaction curves.

Diversity and statistical analysis: Alpha diversity (Shannon index, Chao1 richness, ACE, and observed species) was calculated using Mothur (v1.48.0) and compared between groups using the Wilcoxon rank-sum test with Benjamini-Hochberg false discovery rate (FDR) correction. Beta diversity was assessed using principal coordinate analysis (PCoA) based on Bray-Curtis dissimilarity matrices, with group differences tested by analysis of similarity (ANOSIM) using 999 permutations. Differential abundance of taxa at phylum and genus levels was evaluated using an unpaired two-tailed Student’s *t*-test (for normally distributed data) or Mann-Whitney U test (for non-normal data), with *p* < 0.05 considered statistically significant. All statistical analyses were performed in R (v4.2.1) using the “vegan” and “phyloseq” packages.

### 4.10. Real-Time Fluorescent PCR

Total RNA was isolated from mouse midbrain tissue using TRIzol™ reagent (Thermo Fisher Scientific, Shanghai, China) according to the manufacturer’s instructions. Subsequently, 1 μg of total RNA was reverse transcribed into cDNA using the PrimeScript™ RT Reagent Kit with gDNA Eraser (Takara Bio Inc., Kusatsu, Japan) following the provided protocol. Quantitative real-time PCR (qRT-PCR) was performed on a StepOnePlus™ Real-Time PCR System (Applied Biosystems, Waltham, MA, USA). Gene expression levels were normalized to the endogenous reference gene β-actin. Amplicon specificity was validated by analysis of melting curves generated at the end of each qPCR run. Relative gene expression quantification was performed using the comparative threshold cycle (ΔΔCq) method. Primer sequences and their corresponding annealing temperatures are detailed in Support Information Table S1.

### 4.11. Protein Immunoblotting Experiments

Protein expression levels of α-syn and TH were assessed by Western blotting, with normalization to β-actin as a loading control. All procedures were conducted on ice to minimize proteolytic degradation. Midbrain tissue samples were homogenized in ice-cold RIPA lysis buffer supplemented with 1 mM PMSF and protease inhibitor cocktail. After homogenization, samples were incubated on ice for 30 min to ensure complete lysis. Subsequently, lysates were subjected to brief sonication on ice and then clarified by centrifugation at 12,000× *g* for 15 min at 4 °C. The resulting supernatants were collected as total protein extracts. Protein concentration was quantified using the bicinchoninic acid assay kit (BCA Protein Assay Kit, Solarbio, Beijing, China). After separation by sodium dodecyl sulfate-polyacrylamide gel electrophoresis, proteins were electrophoretically transferred onto polyvinylidene fluoride membranes. The membranes were subsequently blocked with 5% non-fat dry milk in Tris-buffered saline with Tween 20 for 1 h at room temperature. Following blocking, membranes were incubated overnight at 4 °C with the following primary antibodies diluted in blocking buffer: TH and α-syn. Following three washes with TBST (10 min each), the membranes were incubated with an appropriate HRP-conjugated secondary antibody for 1 h at room temperature. After extensive washing to remove unbound antibody, immunoreactive bands corresponding to target proteins were visualized using enhanced chemiluminescence substrate. Band intensities for target proteins were quantified using Image-Pro Plus 6.0 software and normalized to the corresponding β-actin band intensities within each sample.

### 4.12. Statistical Analysis

All statistical analyses were performed using R (v4.2.1), GraphPad Prism (v10.0.2), and SPSS (v28.0). Details specific to each data type are provided below. A summary of statistical methods, including multiple testing corrections

#### 4.12.1. General Data Processing and Group Comparisons

Quantitative data is expressed as mean ± standard error of the mean (SEM). For comparisons between two groups (PFAS-exposed vs. control), an unpaired two-tailed Student’s *t*-test was used when data met normality (Shapiro-Wilk test, *p* > 0.05) and homogeneity of variance (Levene’s test, *p* > 0.05) assumptions. When normality or variance homogeneity assumptions were violated, the Mann-Whitney U test was used instead. For all general comparisons (behavioral tests, qRT-PCR, and protein expression), statistical significance was defined as *p* < 0.05 without multiple testing correction, as these were targeted confirmatory analyses testing pre-specified hypotheses. For multi-omics data (transcriptomics and metabolomics), Benjamini-Hochberg False Discovery Rate (FDR) correction was applied to account for multiple testing, with an adjusted *p*-value < 0.05 considered statistically significant.

#### 4.12.2. Transcriptomics Data Processing and Multiple Testing Correction

Differential expression analysis was performed using DESeq2 (v1.36.0) as described in Section 2.7.2. To control for false positives due to multiple comparisons (315 genes tested), the Benjamini-Hochberg false discovery rate (FDR) procedure was applied. Differentially expressed genes (DEGs) were defined as those with FDR < 0.05 and |log_2_ fold change| ≥ 1.5 (equivalent to a 1.5-fold change in either direction). These thresholds were chosen to balance discovery of biologically relevant changes with control of type I error.

#### 4.12.3. Metabolomics Data Processing and Multiple Testing Correction

Orthogonal partial least squares-discriminant analysis (OPLS-DA) was performed using the ropls package (v1.30.0) with 7-fold cross-validation. Variable importance in projection (VIP) scores were calculated to rank metabolite contribution to group separation. Differentially abundant metabolites (DAMs) were identified using a combination of: (i) VIP > 1.0, (ii) *p* < 0.05 from Student’s *t*-test, and (iii) Benjamini-Hochberg FDR < 0.05. A total of 130 metabolites met these criteria.

#### 4.12.4. 16S rRNA Data Processing and Multiple Testing Correction

Alpha diversity metrics (Shannon, Chao1, ACE, observed species) were calculated using Mothur (v1.48.0). Intergroup differences in alpha diversity were assessed using the Wilcoxon rank-sum test with Benjamini-Hochberg FDR correction for four comparisons (FDR < 0.05 considered significant). Beta diversity was assessed using principal coordinate analysis (PCoA) based on Bray-Curtis dissimilarity, with group differences tested by analysis of similarity (ANOSIM; 999 permutations). Differential abundance of taxa at phylum and genus levels was evaluated using an unpaired two-tailed Student’s *t*-test (or Mann-Whitney U test when assumptions were violated) with Benjamini-Hochberg FDR correction applied separately to phylum-level (10 comparisons) and genus-level (20 comparisons) analyses.

#### 4.12.5. qRT-PCR Validation

qRT-PCR data were analyzed using the comparative threshold cycle (ΔΔCq) method with β-actin as the endogenous reference. Group comparisons were performed using an unpaired two-tailed Student’s *t*-test (*n* = 6 per group). Because these analyses targeted specific genes (*Dbh*, *Tyrp1*, *Got1l1*, *Htr5b*, and *Gng8*) selected based on RNA-seq findings and pre-existing literature, no multiple testing correction was applied (*p* < 0.05 was considered significant). This approach is consistent with targeted validation studies.

## 5. Conclusions

**Figure 18:**
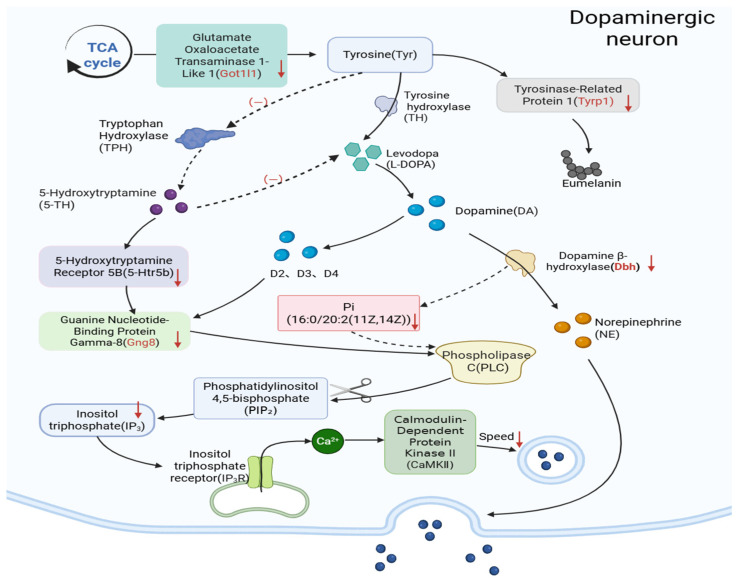
Hypothesized model (correlative, not causally validated) of midbrain dopaminergic neuron changes associated with PFAS mixture exposure. Solid arrows represent observed correlations; dashed arrows represent hypothesized relationships requiring functional validation. Red genes: expression verified by qRT-PCR.

Based on the findings of the present study and an integrated analysis of gene expression and serum metabolite alterations, we elucidated potential mechanisms by which co-exposure to PFASs induces neurotoxicity in midbrain dopaminergic neurons, as detailed in Figure 17. Our findings reveal correlations between PFAS mixture exposure, transcriptomic changes in TM/5-HT-related genes (e.g., *Dbh*, *Tyrp1*, *Htr5b*), and metabolomic alterations in calcium-related lipids. These observations support the hypothesis that PFAS may affect dopaminergic neurons via TM/5-HT and calcium-related pathways, but direct causal evidence and functional validation remain necessary. Within the TM pathway, downregulation of *Tyrp1* expression is postulated to decrease the diversion of tyrosine toward melanin synthesis, thereby increasing Tyr availability for dopamine production. Furthermore, within the 5-HT pathway, scarcity of tyrosine limits tryptophan availability, indirectly suppressing tryptophan hydroxylase activity and reducing 5-HT synthesis. Moreover, the synergistic downregulation of *Htr5b* and *Gng8* compromises the efficiency of receptor-G protein coupling. The convergent impact of dual-pathway dysregulation manifests as three core pathological consequences: (1) impaired TCA cycle flux compromises mitochondrial energy metabolism; (2) diminished Ca^2+^ release and inhibition of CaMKII function lead to calcium homeostasis dysregulation; and (3) deficits in neurotransmitter synthesis coupled with impaired receptor signaling synergistically disrupt synaptic plasticity. Our findings reveal correlative associations between PFAS exposure and alterations in TM/5-HT pathway genes (e.g., *Dbh*, *Tyrp1*, *Htr5b*) and metabolites (e.g., PI(16:0/20:2)). These associations suggest that PFAS may affect dopaminergic neurons via TM/5-HT and calcium-related pathways. However, causality has not been established. The proposed model (Figure 18) is a hypothesis-generating framework that requires functional validation through pharmacological, genetic, or rescue experiments.

## Data Availability

The original contributions presented in this study are included in the article/Supplementary Materials. Further inquiries can be directed to the corresponding author.

## Supplementary Materials

The following supporting information can be downloaded at: https://www.mdpi.com/article/10.3390/ijms27104543/s1.
