# Supplementary material for: Multi-Omics Integrated Analysis Reveals Correlative Signatures of Short-Chain PFAS Mixtures on Mouse Midbrain Dopaminergic Neurons Involving the TM/5-HT Pathway
Source: Int J Mol Sci. 2026 May 19;27(10):4543. doi: 10.3390/ijms27104543 (PMC13207523; doi:10.3390/ijms27104543)
Supplement: Supplementary file 1 [file ijms-27-04543-s001.zip › ijms-4232950-supplementary.pdf]

Support Information

Table 1 Sequence of PCR primer

Primer name	Primer sequence (5'-3')	Clip Length (bp)
β-actin-F	GGCTGTATTCCCCTCCATCG	154
β-actin-R	CCAGTTGGTAACAATGCCATGT	
TH-F	GTCTCAGAGCAGGATACCAAGC	179
TH-R	CTCTCCTCGAATACCACAGCC	
α-syn-F	GCAAGGGTGAGGAGGAGGGTA	90
α-syn-R	CCTGGACTCTCTTTTGGGCTTTA	
Dbh-F	GAGGCGGCTTCCATGTACG	113
Dbh-R	TCCAGGGGGATGTGGTAGG	
*Tyrp1*_F	CCCCTAGCCTATATCTCCCTTTT	229
*Tyrp1*_R	TACCATCGTGGGGATAATGGC	
*Got1l1*_F	CCACGGGTGCTCGGATAATC	162
*Got1l1*_R	TGATGTGATCCCACGACCCA	
*Htr5b*_F	TTGCTGATCGCTGCCACTTT	101
*Htr5b*_R	GTCGAGGCCACCAAGTTATGT	
*Gng8*_F	TCGCATGAAGGTGTCGCAG	115
*Gng8*_R	CTTGTCGCGGAAGGGATTCTC	

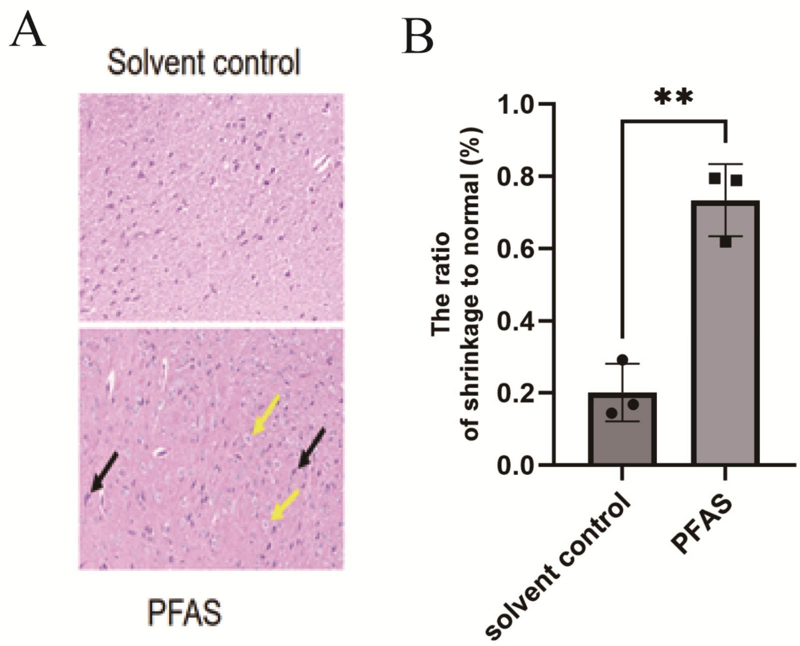

Figure.S1 HE staining results（200×）

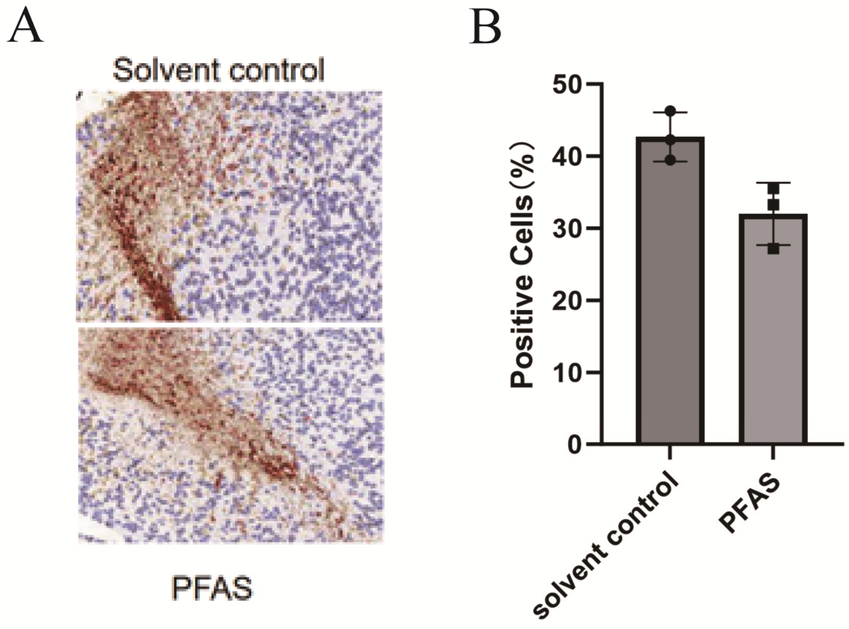

Figure.S2 Immunohistochemical test results

Note：A. TH^{+} neurons in substantia nigra (40×), The brownish-yellow part represents

TH^{+} neurons; B: TH^{+} cell count ratio of substantia nigra.

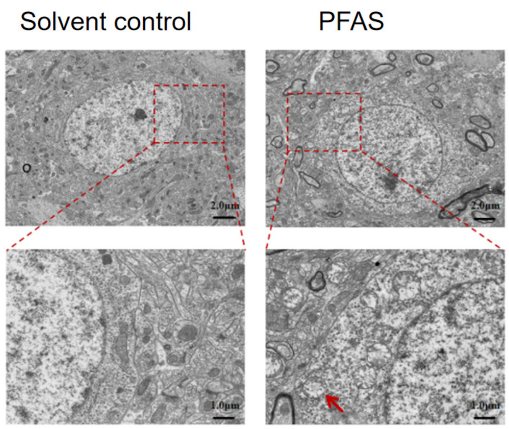

Figure.S3 Transmission electron microscope experimental results

Note: The ruler lines in the figure represent 2μm and 1μm, respectively.

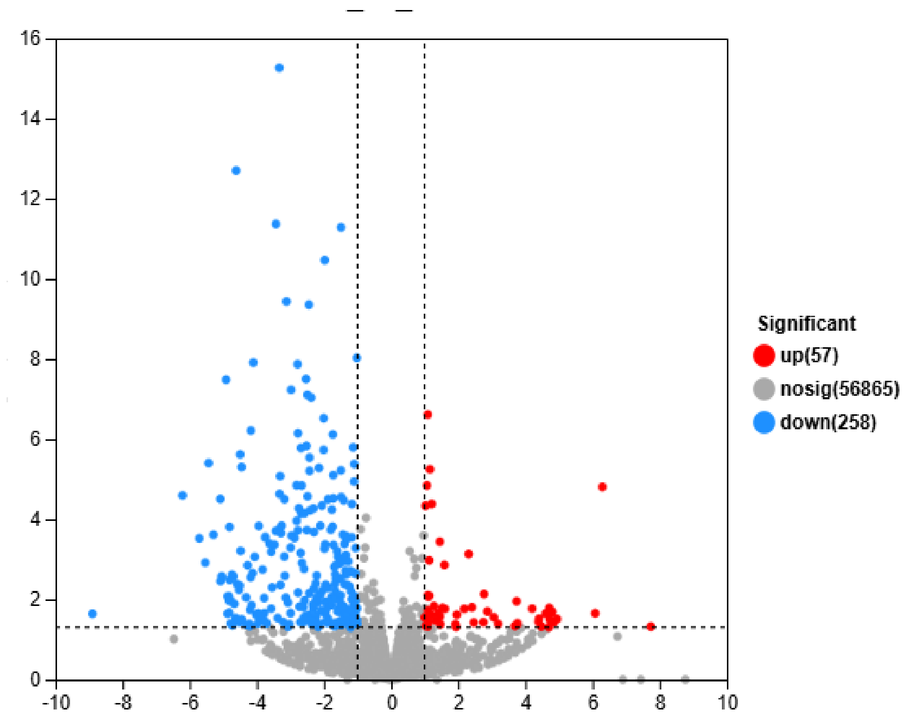

Figure.S4 Volcano plot showing the DEGs

Note: Red dots represent upward DEGs, and blue dots represent downward DEGs.

Red = upregulated, blue = downregulated, gray = not significant.

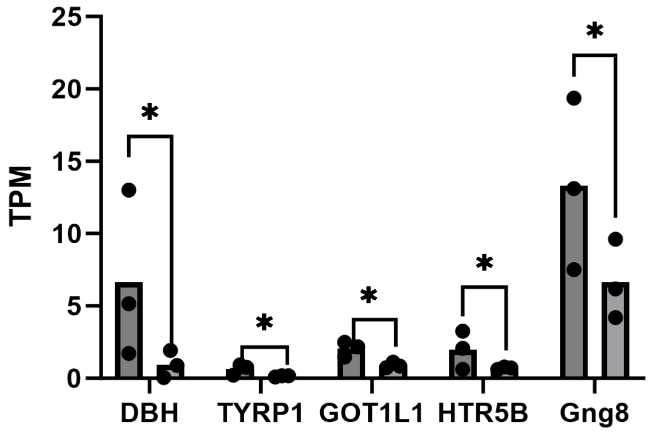

Figure.S5Bar chart of the expression level of the target gene

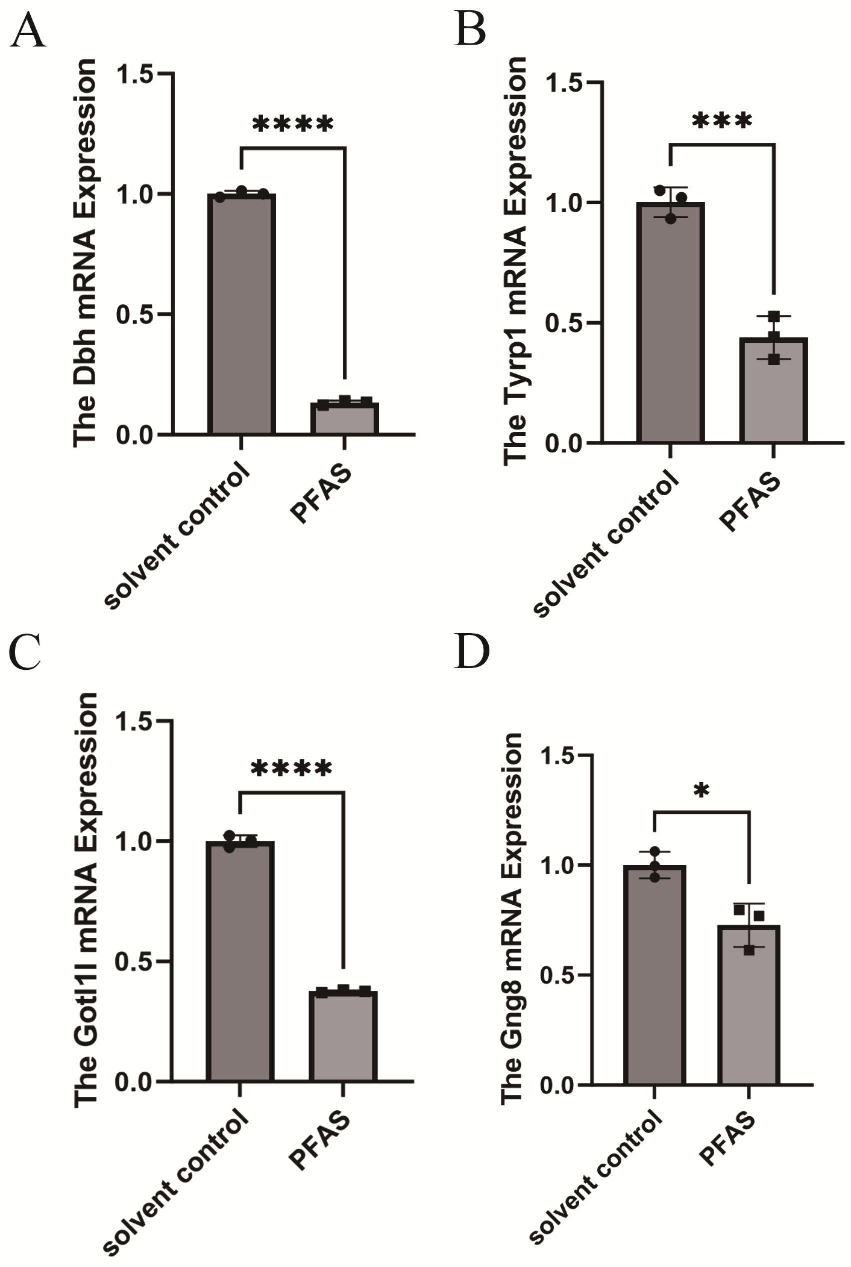

Figure.S6 Changes in the expression of target genes

Note: (A) Relative expression of *Dbh* mRNA; (B) The relative expression of *Tyrp1* mRNA; (C) The relative expression level of *Got1l1* mRNA; (D) The relative expression level of *Gng8* mRNA; "*"means $P<0.05$ compared with Solvent control group.

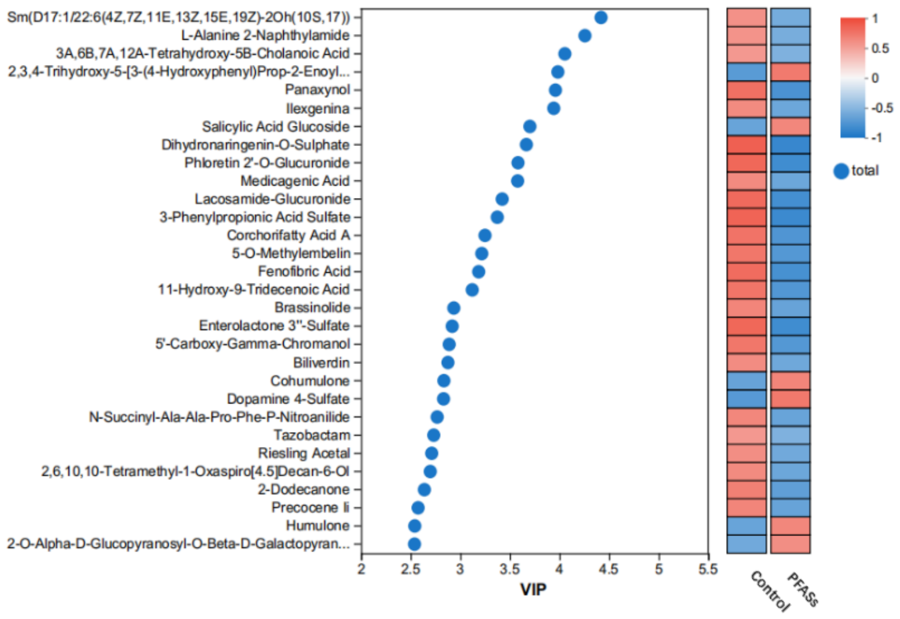

Figure.S7 VIP value Stream Mapping

Note: The left and right two pieces, respectively, represent the solvent control group and the PFASs group. Red indicates that the expression level of metabolites is high in this group, and blue indicates that the expression level of metabolites is low. red = high expression, blue = low expression

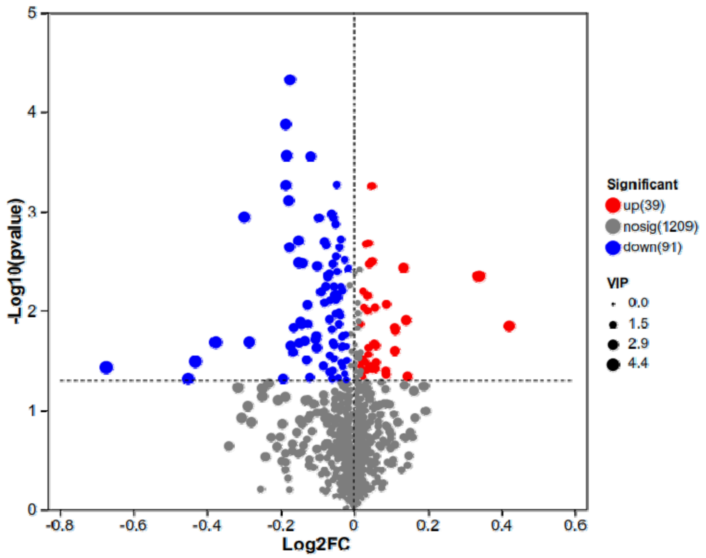

Figure.S8 DMs volcano plot

Note: Red dots indicate high levels of metabolite expression, while blue dots indicate low levels of metabolite expression.

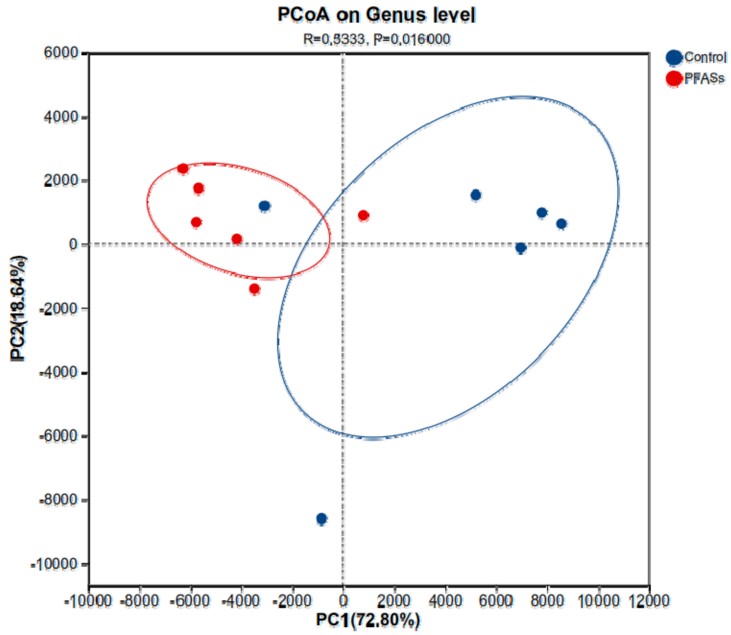

Figure.S9 PCoA analysis
